# Mitochondria Energy Metabolism Depression as Novel Adjuvant to Sensitize Radiotherapy and Inhibit Radiation Induced‐Pulmonary Fibrosis

**DOI:** 10.1002/advs.202401394

**Published:** 2024-05-07

**Authors:** Zaigang Zhou, Xin Jiang, Lei Yi, Cheng Li, Haoxiang Wang, Wei Xiong, Zhipeng Li, Jianliang Shen

**Affiliations:** ^1^ National Engineering Research Center of Ophthalmology and Optometry Eye Hospital Wenzhou Medical University Wenzhou 325027 China; ^2^ Department of Urology, Xiangya Hospital Central South University Changsha Hunan 410008 China; ^3^ Department of Urology The Second Xiangya Hospital, Central South University Changsha Hunan 410011 China; ^4^ Department of Urology The Third Xiangya Hospital, Central South University Changsha Hunan 410013 China; ^5^ Zhejiang Engineering Research Center for Tissue Repair Materials Wenzhou Institute University of Chinese Academy of Sciences Wenzhou Zhejiang 325001 China

**Keywords:** mitochondria metabolism regulation, programmed death ligand‐1, radiation induced‐pulmonary fibrosis, radioimmunotherapy, transforming growth factor‐β

## Abstract

Currently, the typical combination therapy of programmed death ligand‐1 (PD‐L1) antibodies with radiotherapy (RT) still exhibits impaired immunogenic antitumor response in clinical due to lessened DNA damage and acquired immune tolerance via the upregulation of some other immune checkpoint inhibitors. Apart from this, such combination therapy may raise the occurrence rate of radiation‐induced lung fibrosis (RIPF) due to enhanced systemic inflammation, leading to the ultimate death of cancer patients (average survival time of about 3 years). Therefore, it is newly revealed that mitochondria energy metabolism regulation can be used as a novel effective PD‐L1 and transforming growth factor‐β (TGF‐β) dual‐downregulation method. Following this, IR‐TAM is prepared by conjugating mitochondria‐targeted heptamethine cyanine dye IR‐68 with oxidative phosphorylation (OXPHOS) inhibitor Tamoxifen (TAM), which then self‐assembled with albumin (Alb) to form IR‐TAM@Alb nanoparticles. By doing this, tumor‐targeting IR‐TAM@Alb nanoparticle effectively reversed tumor hypoxia and depressed PD‐L1 and TGF‐β expression to sensitize RT. Meanwhile, due to the capacity of heptamethine cyanine dye in targeting RIPF and the function of TAM in depressing TGF‐β, IR‐TAM@Alb also ameliorated fibrosis development induced by RT.

## Graphical abstract

In this research, IR‐TAM is prepared by conjugating heptamethylene cyanine with Tamoxifen to lower the dosage of Tamoxifen needed in inhibiting PD‐L1 and TGF‐β expression. Then, IR‐TAM can self‐assembly with albumin to form IR‐TAM@Alb nanoparticles. By doing this, IR‐TAM@Alb can enhance radio‐immunotherapy and attenuate radiation induced‐pulmonary fibrosis through simultaneously targeting of TGF‐β and PD‐L1.

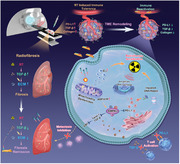

## 1 Introduction

At present, radiotherapy (RT) is widely used as the palliative or curative treatment of a good deal of solid tumors like bladder cancers and breast cancers.^[^ ^1^ ^]^ It has been postulated that immune priming or activation induced by localized RT may facilitate the transformation of immune‐cold tumors into inflamed‐hot tumors by eliciting numerous positive effects on the tumor microenvironment (TME), including inducing immunogenic cell death, upregulating antigen presentation, and enhancing leukocyte influx.^[^ ^2^ ^]^ But it's still a pity that the efficacy of RT in activating the tumor immunity was severely impaired due to the poor tumor microenvironment like hypoxia, which decreased the amount of DNA damage and reactive oxygen species (ROS).^[^ ^2^, ^3^ ^]^ Besides, the immune tolerance after RT such as increased expression of programmed death ligand‐1(PD‐L1) and transforming growth factor‐β (TGF‐β) proteins would also impair the efficacy of RT in converting immune‐cold tumors into inflamed‐hot tumors by decreasing the activation of T cells and amplifying the distribution of T regulatory cells in tumors.^[^ ^4^ ^]^ Last but not least, due to the systemic inflammation induced by RT, radiation‐induced lung fibrosis (RIPF) always occurs (≈50%) due to fibroblast activation, leading to impaired lung function and ultimate death of cancer patients owing to lung failure, which was often overlooked in the designing of safe and effective RT sensitizer.^[^ ^5^ ^]^ Thus, novel and highly efficient drugs or nanosystems that could establish perfect TME to sensitize RT and prevent or cure radiation‐induced pulmonary fibrosis simultaneously are still urgently needed.

At present, a lot of PD‐L1 or Programmed death receptor 1 (PD‐1) antibodies like Navulizumab, Pabolizumab, and Duvarizumab were used to block the recognition of tumor cell membrane located PD‐L1 and T cell membrane located PD‐1, which was believed to prevent the acquired immune resistance induced by the upregulated PD‐L1 expression after RT.^[^ ^6^ ^]^ However, as newly proven, the cytoplasm and nucleus located‐PD‐L1 also played vital roles in the occurrence, development, and deterioration of tumors.^[^ ^7^ ^]^ It was interesting that the cytoplasm located PD‐L1 could speed up the DDR process by the stabilization of DDR‐related messenger RNA (mRNAs) like NBS1, MRE11, and RAD50.^[^ ^7^, ^8^ ^]^ By doing this, the efficacy of DNA‐damaging therapies like RT and platinum drug‐based chemotherapy was impaired.^[^ ^7^, ^8^ ^]^ Apart from this, the perinuclear or intracellular located PD‐L1 can bind to DNA‐dependent protein kinases to activate mitogen‐activated protein kinase or extracellular regulated protein kinases, which then promotes the cell survival signaling to maintain the growth of tumor cells.^[^ ^7^, ^9^ ^]^ Thus, the efficacy of clinically used PD‐L1 antibodies in sensitizing RT is very limited since most PD‐L1 antibodies could only impair the function of tumor cell membrane located‐PD‐L1 protein rather than affect the function of cytoplasm and nucleus located‐PD‐L1 simultaneously.^[^ ^7^, ^10^ ^]^ Meanwhile, no success of PD‐L1 antibodies in inhibiting the TGF‐β pathway made the occurrence and development of RIPF unaffected.^[^ ^11^ ^]^ Moreover, some avoid‐less defects of anti‐PD‐L1/PD‐1 monoclonal antibodies like too high cost, systemic immunogenicity risk, limited tumor tissue accumulation, poor efficacy, and no‐tumor targeting capacity would further limit their combination therapy with RT.^[^ ^7^, ^12^ ^]^ To sum up, the currently used PD‐1/PD‐L1 could not fulfill the requirements of the highly efficient RT combination therapy both in clinical and basic research.

Recently, tumor mitochondria metabolism regulation was newly revealed to play a vital role in the deterioration of TME, which may maintain the immune tolerance of non‐inflamed cold TME with increased PD‐L1 and TGF‐β expression.^[^ ^13^ ^]^ As we all know, most solid tumors exhibit increased adenosine triphosphate (ATP) generation by raising oxidative phosphorylation (OXPHOS) and glycolysis levels.^[^ ^13^, ^14^ ^]^ Due to this, the adenosine diphosphate ADP/ATP ratio was decreased, leading to the following depressed phosphorylation of Adenosine 5′‐monophosphate (AMP)‐activated protein kinase (AMPK) protein.^[^ ^13^, ^15^ ^]^ Then, activated AMPK directly bound to PD‐L1 and phosphorylated PD‐L1 at S195, resulting in its endoplasmic reticulum accumulation and the following endoplasmic reticulum‐associated degradation of the PD‐L1 protein.^[^ ^13^ ^]^ Apart from this, the secretion of TGF‐β from tumor cells could also be inhibited by the increased AMPK phosphorylation, which then led to immune activation, as well as the following possibly reversed fibroblast activation and fibrosis development.^[^ ^16^ ^]^ Thus, OXPHOS or glycolysis inhibition may serve as a versatile strategy to sensitize RT via hypoxia reversion, PD‐L1, and TGF‐β depression, as well as RIPF attenuation. But, whether this strategy could work as expected had yet to be confirmed since little attention noticed the potential of this strategy.

For the past decades, Tamoxifen (TAM) has been used as the most widely used adjuvant chemotherapeutic for the endocrine treatment of almost all stages of estrogen receptor (ER)‐positive breast cancer, which significantly extended the survival time or even disease‐free survival of millions of women, especially for postmenopausal patients.^[^ ^17^ ^]^ Interestingly enough, a large body of preclinical or clinical data also indicated that TAM could modulate multiple cellular processes like activation AMPK pathway through OXPHOS depression by inhibiting mitochondria complex I independently of ER status.^[^ ^18^ ^]^ Thus, theoretically, TAM may possess the potential for new use of old drugs as PD‐L1 and TGF‐β immune‐pathway dual‐depression agents to sensitize tumor immunotherapy like RT.^[^ ^18^, ^19^ ^]^ However, whether TAM possesses such capacity to inhibit PD‐L1 and TGF‐β expression, as well as remising RIPF still needs to be proved.^[^ ^19^ ^]^ Apart from this, as an adjuvant chemotherapeutic, TAM possessed no tumor‐targeting or fibrosis‐targeting capacity, which then at least partly was the cause of the failure and acquired resistance of TAM therapy.^[^ ^18^ ^]^ Thus, targeted delivery of TAM to the tumors and fibrosis may serve as a novel strategy to better convert cold tumors to hot ones after RT, as well as prevent or attenuate RIPF.

**Scheme 1:**
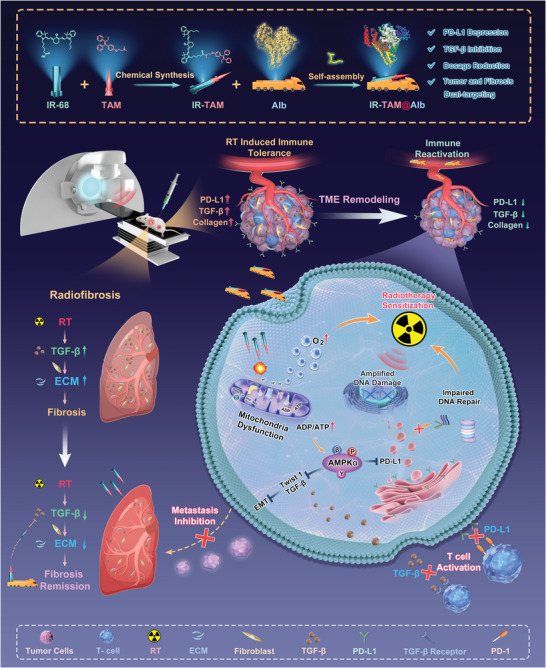
The preparation process of IR‐TAM@Alb nanoparticles and the mechanism underlying their enhanced radio‐immunotherapy and attenuated RIPF through simultaneously targeting of TGF‐β/PD‐L1.

As is well known, some heptamethine cyanine dyes possessed ideal tumor mitochondria targeting capacity.^[^ ^20^ ^]^ Besides, it was also newly revealed that some heptamethine cyanine dye could also selectively accumulate at the fibrosis area.^[^ ^21^ ^]^ Moreover, conjugating TAM with mitochondria‐targeting agents may also decrease the dosage of TAM needed to induce mitochondria dysfunction.^[^ ^16^ ^]^ Thus, conjugating TAM with heptamethine cyanine dye may be used as a tumor and fibrosis dual‐targeting and co‐therapy agent for effective RT and fibrosis prevention by PD‐L1 and TGF‐β depression. To prove this speculation, we designed IR‐TAM by conjugating mitochondria‐targeted heptamethine cyanine dye IR‐68 with mitochondrial complexes I depression agent TAM and then further self‐assembled with albumin (Alb) to form IR‐TAM@Alb nanoparticles (**Scheme** **1**). By doing this, IR‐TAM@Alb nanoparticles rather than TAM possessed ideal tumor targeting capacity to depress PD‐L1 and TGF‐β expression (TAM 30 µm versus IR‐TAM 4 µm), which then sensitized tumor RT by enhancing DNA damage, amplifying T cell infiltration, and preventing the possible acquired immune resistance induced by RT. Apart from this, due to the fibrosis‐targeting capacity of IR‐TAM@Alb, IR‐TAM@Alb better accumulated in the fibrosis area to depress RIPF by reducing TGF‐β secretion, collagen distribution, and fibronectin secretion (Scheme 1). To sum up, in this study, we innovatively designed a tumor/fibrosis dual‐targeting and co‐therapy strategy to eradicate RT‐resistant tumors while sparing normal lung tissues, making up for the gaps in clinical and basic research (Scheme 1).

## 2 Results

### 2.1 IR‐TAM Downregulated PD‐L1 and TGF‐β Expression via AMPK Pathway Activation

**Figure 1:**
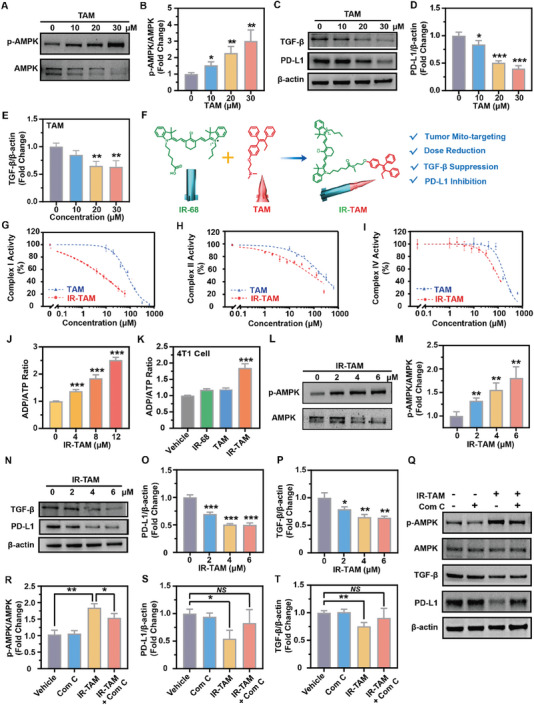
IR‐TAM more effectively inhibited the expression of PD‐L1 and TGF‐β1 by the activation of the AMPK pathway through mitochondrial function inhibition. A–E) Detection of p‐AMPK, TGF‐β1, and PD‐L1 expression in 4T1 cells by western blotting assay after treatment with gradient concentrations of TAM for 24 h and further quantified by ImageJ (*n* = 3). F) Schematic diagram of the synthesis process of IR‐TAM and its improved functions compared with TAM. G–I) Detection of mitochondria complex I, II, and IV activities after various concentrations of TAM or IR‐TAM treatment (*n* = 3). J,K) Evaluation of the ratio of ADP/ATP in 4T1 cells after treatment of gradient concentrations of IR‐TAM or different groups of Vehicle, IR, TAM, and IR‐TAM (*n* = 3). L–P) Evaluation of the expression of p‐AMPK, PD‐L1, and TGF‐β protein in 4T1 cells by western bolt assay after treatment by indicated concentrations of IR‐TAM, and quantified with ImageJ. Q–T) The expression of p‐AMPK, PD‐L1, and TGF‐β protein after IR‐TAM treatment with or without the addition of 10 µm Compound C and corresponding quantitative analysis. Data were demonstrated as mean ± SD. * *p* < 0.05, ** *p* < 0.01, and *** *p* < 0.001.

Recent studies have demonstrated that TAM, an FDA‐approved antiestrogen medication indicated for the treatment of patients diagnosed with ER‐positive breast cancer, can also disrupt mitochondrial metabolism in an ER‐independent manner, specifically by inhibiting complex I of the respiratory chain and thereby reducing cellular oxygen consumption.^[^ ^18^ ^]^ Then, it activated the AMPK pathway, a key regulator of cellular energy homeostasis, which in turn may modulate the degradation of ER‐related proteins such as PD‐L1 and TGF‐β via AMPK phosphorylation.^[^ ^4^, ^13^, ^22^ ^]^ However, whether TAM possessed the desired PD‐L1 and TGF‐β depression capacity still needed to be revealed.^[^ ^18^ ^]^ In this study, we first assessed the impact of TAM on p‐AMPK, PD‐L1, and TGF‐β expression in 4T1 mouse breast cancer cells (**Figure** **1**A–E). Our findings indicated that a high dosage of TAM (30 µm) could activate AMPK, which then partially inhibited the expression of PD‐L1 and TGF‐β in these cells (**Figure** **1**A–E). Thus, TAM may be used as a novel dual‐immune checkpoint inhibitor via PD‐L1 and TGF‐β inhibition, which may further expand its clinical application in the future (Figure 1A–E).

However, even so, TAM's hydrophobic characteristic, high effective dose, and lack of tumor mitochondrial targeting capacity would still result in its’ poor tumor response and systemic side effects, thereby limiting its clinical potential benefits.^[^ ^16^, ^18^ ^]^ To solve this dilemma, IR‐TAM was prepared by the chemical group modification of TAM by mitochondria‐targeted heptamethine cyanine dye IR‐68 was used to enhance its tumor mitochondria targeting capacity, so as to enhance the inhibition of mitochondrial aerobic respiration of TAM to activate AMPK pathway more efficiently, thus to play a downstream regulatory role (Figure 1F; Figure S1, Supporting Information). The HR‐MS, ^1^H‐NMR, and ^13^C‐NMR spectra confirmed the accurate synthesis and preparation of the novel compound IR‐TAM (Figures S2–S5, Supporting Information). Then, to evaluate whether IR‐TAM could more effectively affect aerobic respiration, mitochondrial respiratory chain complex I, II, and IV inhibition assays were performed (Figure 1G–I). Results showed that IR‐TAM could inhibit Complex I at a very low dosage, while TAM could only realize such function at a very high dosage (Figure 1G–I). Subsequently, inhibition of aerobic respiration led to changes in the ADP/ATP ratio, which was a crucial prerequisite for AMPK pathway activation.^[^ ^4^, ^17^ ^]^ Therefore, we measured the ADP/ATP ratio and found that IR‐TAM effectively increased the ADP/ATP ratio in a dose‐dependent manner and was also more effective than synthetic components such as IR‐68 and TAM (Figure 1J–K).

After confirming the superior efficacy of IR‐TAM in inhibiting mitochondrial function, we proceeded to assess its regulatory potential on AMPK, PD‐L1, and TGF‐b in 4T1 cells (Figure 1L–P). Consistent with the proven mitochondrial inhibition effect above, IR‐TAM exhibited a greater ability to activate the AMPK pathway and inhibited PD‐L1 and TGF‐β expression compared to TAM, while requiring a lower dosage (TAM 30 µm versus IR‐TAM 4 µm) (Figure 1L–P). Interestingly enough, high‐dosage IR‐68 did not possess such a function as IR‐TAM in depressing PD‐L1 and TGF‐β expression (Figures S6 and S7, Supporting Information). Recent studies have shown that one vital relevant mechanism for downregulating PD‐L1 expression was through the degradation of ER‐associated proteins, which was mediated by AMPK phosphorylation.^[^ ^13b,c^ ^]^ To assess whether IR‐TAM‐induced PD‐L1 and TGF‐β down‐regulation was also mediated by AMPK phosphorylation, Compound C (Com C, an AMPK inhibitor) was used.^[^ ^13^, ^14^ ^]^ As results indicated, Com C reversed IR‐TAM‐induced AMPK phosphorylation, thereby counteracting IR‐TAM mediated down‐regulation of PD‐L1 and TGF‐β in 4T1 cells (Figure 1Q–T). All in all, IR‐TAM rather than TAM or IR‐68 more effectively downregulated the PD‐L1 and TGF‐β expression via AMPK pathway activation.

### 2.2 Preparation and Characterization of IR‐TAM@Alb Nanoparticles

Considering the limitations of IR‐TAM, such as its low water solubility, high toxicity, and aggregation‐induced quenching (ACQ), its application in organisms was restricted, including diagnosis and treatment. Therefore, constructing an appropriate nanosystem can fully exploit the advantages of nanomedicine in vivo, such as regulating drug release rate, increasing biofilm permeability, altering body distribution, and enhancing bioavailability while overcoming the limitations of single drug IR‐TAM. Recent studies have shown that Alb can form nanoparticles by self‐assembly with some heptamethylcyanine dyes.^[^ ^23^ ^]^ Therefore, IR‐TAM with a similar structure may also have a strong affinity with Alb.

**Figure 2:**
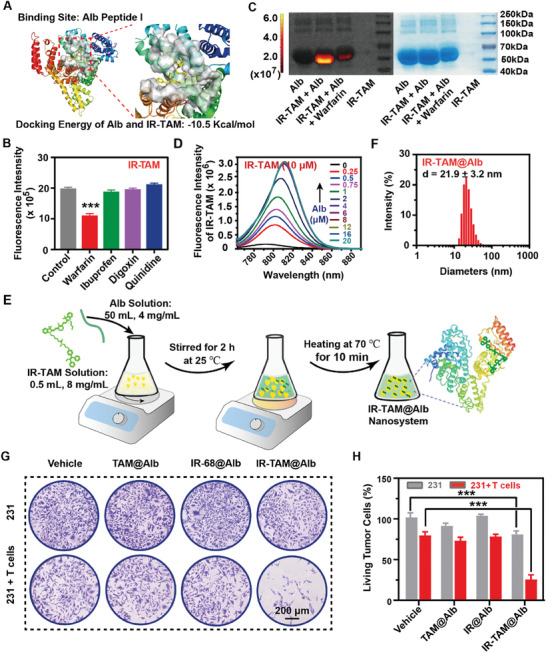
Characterization of the IR‐TAM@Alb nanoparticles and its capacity in promoting the tumor‐killing capacity of T cells in vitro. A) Molecular docking to reveal the binding site of Alb with IR‐TAM. B) The analysis of the Alb‐binding site of IR‐TAM through the competitive inhibitors warfarin, ibuprofen, digoxin, and quinidine in correspondence with Alb‐bond sites I, II, III, and α1‐glycolipoprotein, respectively (*n* = 3). C) The affinity of IR‐TAM with Alb verified by gel electrophoresis and the following detection of IR‐TAM by fluorescence imaging. D) Detection of the fluorescence intensity change of 10 µM IR‐TAM when incubated with various concentrations of Alb. E) The preparation process of IR‐TAM@Alb nanosystem. F) Hydrodynamic diameters distribution of IR‐TAM@Alb nanoparticles in deionized water (*n* = 3). G) Effects of IR‐TAM@Alb on the killing capacity CD8 T cell of MDA‐MB‐231cells. MDA‐MB‐231 cells were treated with TAM@Alb, IR@Alb, or IR‐TAM@Alb for 24 h, and followed by co‐culture with activated CD8 T cell. H) Living MDA‐MB‐231 cells were quantified. Data were demonstrated as mean ± SD. *** *p* < 0.001.

Through molecular docking simulation calculations, we confirmed that IR‐TAM exhibits a favorable affinity toward binding site I of Alb, due to their docking energy of −10.5 Kcal mol^−1^ (**Figure** **2**A). Then, we conducted a further assessment of the albumin‐binding site of IR‐TAM using previously established methods (Figure S8, Supporting Information).^[^ ^23^ ^]^ Competitive inhibitors, including warfarin, ibuprofen, digoxin, and quinidine were employed to target the Alb binding sites I, II, III, and α1‐glycolipoproteins respectively.^[^ ^23^ ^]^ Among these tested inhibitors, warfarin demonstrated a significant reduction in the fluorescence intensity of IR‐TAM, indicating the high‐affinity binding of IR‐TAM to Alb binding site I (Figure 2B). Moreover, the electrophoretic analysis demonstrated the high‐affinity binding of IR‐TAM to the site I and its ability to self‐assemble with Alb to form dye‐Alb complexes, which exhibited significant fluorescence compared to pre‐incubation with warfarin (Figure 2C). In addition, with fixed IR‐TAM concentrations, Alb titration test showed a gradual increase in UV–vis absorption intensity and fluorescence to saturation (Figure 2D; Figure S9, Supporting Information). Due to the proven high‐combination possibility between Alb and IR‐TAM, IR‐TAM@Alb was prepared by previously reported methods (Figure 2E).^[^ ^23^ ^]^ Transmission electron microscopy (TEM) revealed that IR‐TAM@Alb exhibited a spherical morphology with a particle size of ≈20 nm, which was consistent with the dynamic light scattering (DLS) data (Figure 2F; Figure S9, Supporting Information).

### 2.3 IR‐TAM@Alb Nanoparticles Exhibited Tumor Mitochondria Selective Accumulation Behavior In Vitro

Previously, the tumor mitochondrial targeting ability of heptamethine cyanine dye had been confirmed by several studies.^[^ ^23^, ^24^ ^]^ Based on our design regarding the transformation of IR‐TAM by conjugating TAM with IR‐68, we anticipated that IR‐TAM@Alb would also exhibit similar targeting capabilities as IR‐68@Alb (Figure S10, Supporting Information). To investigate the cellular localization relationship between IR‐TAM and mitochondria, a mitochondrial probe was employed (Figure S10, Supporting Information). Results showed that the fluorescence of IR‐TAM@Alb and mitochondrial probe highly coincided in 4T1 cells, indicating the excellent mitochondrial targeting capacity of IR‐TAM@Alb (Figure S10, Supporting Information).

Then, we explored the mechanisms influencing this nanosystem absorption by tumor cells using BSP (a competitive inhibitor of OATPs), chlorpromazine (CPZ, an inhibitor of clathrin‐mediated endocytosis), or at 4 °C (Figures S10 and S11, Supporting Information). Treatment with BSP and culture at 4 °C resulted in the decreased fluorescence derived from IR‐TAM in 4T1 cells, implying that the internalization of IR‐TAM@Alb by tumor cells is contingent upon the presence of organic anion‐transporting polypeptides (OATPs) and the metabolic state of the cells (Figures S10 and S11, Supporting Information).^[^ ^23^, ^24^ ^]^ Next, the viability of the cancer cells after TAM@Alb, IR‐68@Alb, or IR‐TAM@Alb treatment was measured using the CCK‐8 kit. As results showed, IR‐TAM@Alb had a significantly stronger anti‐tumor effect than TAM@Alb on MB49, CT26, and 4T1 tumor cells in vitro (Figure S10, Supporting Information). To sum up, IR‐TAM@Alb nanoparticles exhibited ideal tumor mitochondria selective accumulation behavior and tumor cell killing capacity in vitro.

### 2.4 IR‐TAM@Alb Nanoparticles Enhanced the Cytotoxicity of T Cells to Tumor Cells by Dual‐Inhibition of PD‐L1/TGF‐β

As we proved, IR‐TAM@Alb rather than TAM@Alb or IR‐68@Alb more effectively inhibited PD‐L1/TGF‐β expression in tumor cells (Figure S12, Supporting Information). Therefore, we further investigated whether IR‐TAM@Alb could effectively alleviate the immunosuppressive effect of tumor cells on T cells and enhance their cytotoxicity (Figure 2G). Initially, 5637 and 231 tumor cells were either pre‐treated with TAM@Alb, IR‐68@Alb, or IR‐TAM@Alb, before being co‐cultured with activated T cells (Figure 2G,H). These findings indicated that IR‐TAM@Alb facilitated T cell activation and exerted a more potent cytotoxic effect on tumor cells, whereas TAM@Alb and IR‐68@Alb failed to elicit such responses (Figure 2G,H; Figure S13, Supporting Information). Meanwhile, different groups of T cells were collected after co‐incubation with 5637 cells to detect the mRNA levels of IFN‐γ and IL‐2 in T cells by real‐time quantitative reverse transcription PCR (RT‐qPCR). The results indicated that the mRNA levels of IFN‐γ and IL‐2 in T cells were significantly increased after pre‐treatment with IR‐TAM@Alb (Figure S14, Supporting Information). Thus, IR‐TAM@Alb resulted in the reversal of immunosuppression and enhanced T cell activity in vitro.

### 2.5 IR‐TAM@Alb Nanoparticles Sensitized Radio‐Immunotherapy Through Oxygen Reversion and PD‐L1/TGF‐β Dual‐Inhibition In Vitro

**Figure 3:**
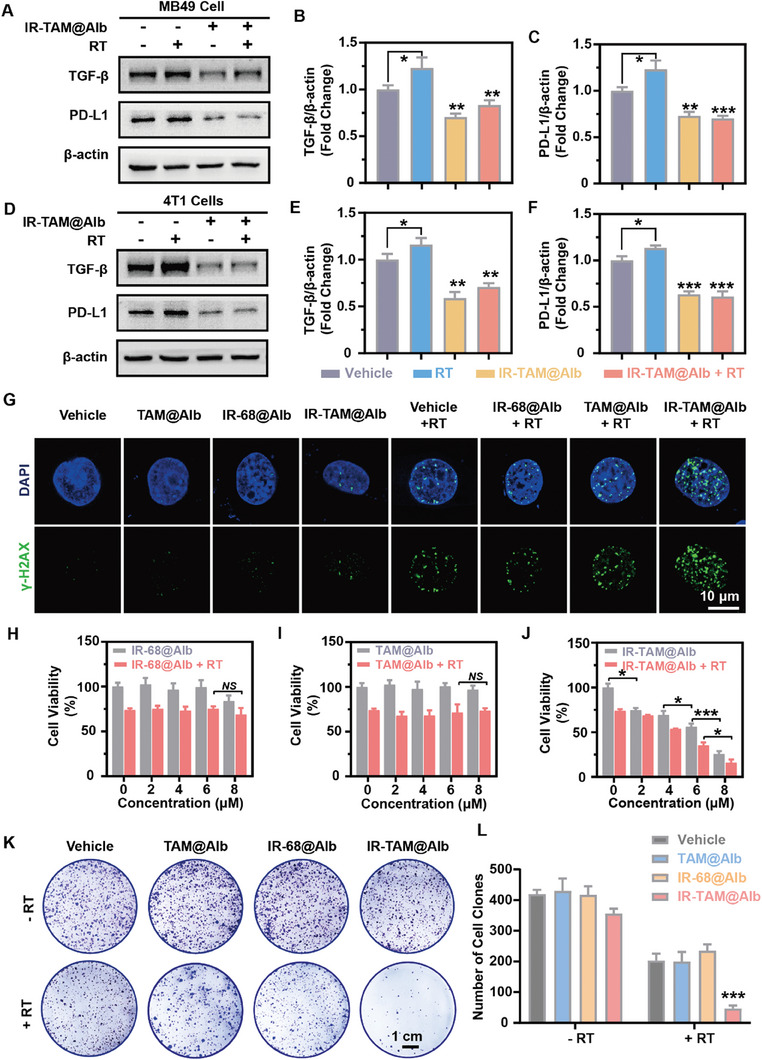
IR‐TAM@Alb enhanced radio‐immunotherapy by inhibiting DNA damage repair and suppressing PD‐L1 and TGF‐β protein expression. (A‐F) Detection of the expression of PD‐L1 and TGF‐β protein in MB49 or 4T1 cells incubated with or without IR‐TAM@Alb for 24 h (IR‐TAM: 6 µm) in the presence or absence of RT (0 or 3 Gy, Efficiency: 6 Gy min^−1^) by western blotting assay. The quantitative analysis of the expression of TGF‐β and PD‐L1 proteins was performed by ImageJ (*n* = 3). G) MB49 cells were treated with vehicle, TAM@Alb, IR‐68@Alb, and IR‐TAM@Alb for 24 h in the absence or presence of RT (3 Gy) subsequently (calculated by TAM, IR‐68, or IR‐TAM concentration: 6 µm). After another 48 h of normal cultivation, cells were subjected to immunofluorescence for investigating the change of γ‐H2 AX in the nucleus (scale bar = 10 µm). H–J) Detection of the cell viability of MB49 cells by CCK‐8 assay after different treatments with various concentrations of TAM@Alb, IR‐68@Alb, or IR‐TAM@Alb with or without RT co‐treatment (3 Gy). K,L) The representative picture of the MB49 cell clones at Day 10 after the pre‐treatment of Vehicle, TAM@Alb, IR@Alb, and IR‐TAM@Alb (calculated by TAM, IR‐68, or IR‐TAM concentration: 6 µm) for 24 h with or without RT co‐treatment (3 Gy) for 48 h (*n* = 3). Data were demonstrated as mean ± SD. * *p* < 0.05, ** *p* < 0.01, and *** *p* < 0.001. “(+ RT)” represents the tumor cells treated with RT. “(‐ RT)” represents the cells treated without laser irradiation. *NS* means no significant difference, compared with the vehicle group.

Although local RT has been shown to stimulate anti‐tumor immune responses, this effect may be partially counteracted by negative factors in the TME.^[^ ^6b^ ^]^ These factors include the induction of angiogenic and fibrogenic growth factors such as TGF‐β, which then increase the tumor extracellular matrix (ECM) density.^[^ ^4^, ^7^ ^]^ Additionally, the enhanced recruitment of immunosuppressive cells and upregulation of immune checkpoints such as PD‐L1 and TGF‐β also occurred.^[^ ^12^, ^13^ ^]^ Therefore, IR‐TAM@Alb had the potential to synergize with RT by simultaneously inhibiting PD‐L1 and TGF‐β, as well as reversing tumor hypoxia (Figure S15, Supporting Information). First, western blot analysis revealed that the expression levels of PD‐L1 and TGF‐β were upregulated in MB49 and 4T1 tumor cells following RT, whereas those cells pre‐treated with IR‐TAM@Alb exhibited a sustained reduction in PD‐L1 and TGF‐β expression compared to the control group (**Figure** **3**A–F). In addition, recent studies revealed that intracellular PD‐L1, as an RNA‐binding protein, protected mRNA related to DNA damage repair from degradation by RNA‐exosome by competing with RNA‐exosome, which led to the compromised efficacy of RT.^[^ ^7^, ^13^ ^]^ Thus, we speculated that IR‐TAM@Alb may impede the DNA damage repair process following RT by down‐regulating the PD‐L1 expression in cells. To validate this hypothesis, we monitored the changes in γ‐H2AX fluorescence foci within MB49 tumor cell nuclei after RT and observed a significant increase of such foci in the IR‐TAM@Alb pre‐treated group compared with other groups subjected to RT (Figure 3G). Subsequently, the cell viability of MB49 cells was assessed using the CCK‐8 assay to investigate the effects of IR‐68@Alb, TAM@Alb, and IR‐TAM@Alb in combination with or without RT (3 Gy). As results indicated, IR‐TAM@Alb could effectively suppress tumor cell activity at a low dosage when combined with radiotherapy (Figure 3H–J). The synergistic anti‐tumor effect of IR‐TAM@Alb was further validated through colony formation assay, showing that the combination of IR‐TAM@Alb and RT exhibited a synergistic effect greater than the sum of their individual effects with the sensitizing enhancement ratios (SER50) of ≈1.56 (Figure 3K,L; Figure S16, Supporting Information). To sum up, IR‐TAM@Alb nanoparticles sensitized RT through oxygen reversion and PD‐L1/TGF‐β dual‐inhibition in vitro.

### 2.6 The Tumor Targeting Capacity of IR‐TAM@Alb Nanoparticles In Vivo

Building upon the remarkable in vitro tumor targeting ability of IR‐TAM@Alb, we proceeded to track the pharmacokinetics of IR‐TAM@Alb in vivo using a live animal imaging system within an MB49 tumor‐bearing model (Figure S17, Supporting Information). As illustrated in Figure S17 (Supporting Information), after intravenous injection of IR‐TAM@Alb, the fluorescence of IR‐TAM at the tumor site gradually increased over time, reaching a peak at ≈24 h. Interestingly, IR‐TAM@Alb appeared to exhibit a prolonged retention time within the tumor (Figure S17, Supporting Information). Subsequently, a part of the mice was sacrificed at 24 h, 48 h, and 72 h to collect the major organs and tumors. Then, the fluorescence analysis was performed to further investigate the precise spatiotemporal distribution of drugs within the organism, showing that IR‐TAM@Alb exhibited the highest mean fluorescence distribution compared to any other organs (Figure S17, Supporting Information). These results altogether suggested that the self‐assembled IR‐TAM@Alb nanoparticles exhibited excellent tumor‐targeting capacity in vivo.

Following this, to assess the toxic side effects of IR‐TAM@Alb in vivo, blood was collected from mice to detect hemolysis, indicating that IR‐TAM@Alb did not possess any ability to induce blood hemolysis (Figure S18, Supporting Information). Besides, after IR‐TAM@Alb treatment for 24 h or 14 days, the kidney and liver function was not affected since the expression levels of creatinine (CRE), aspartate aminotransferase (AST), alanine aminotransferase (ALT), and blood urea nitrogen (BUN) were nearly unchanged compared with the control group (Figure S19, Supporting Information). Moreover, results of the H&E staining of the heart, liver, spleen, lung, and kidney altogether further confirmed that IR‐TAM@Alb possessed no obvious toxicity to normal tissues (Figure S20, Supporting Information). All in all, IR‐TAM@Alb rather than PD‐L1 monoclonal antibodies may better selectively and safely block PD‐1/PD‐L1 recognition and inhibit TGF‐β secretion.

### 2.7 IR‐TAM@Alb Nanoparticles Relieved T‐Cell Exhaustion via Suppressing PD‐L1 and TGF‐β Expression

**Figure 4:**
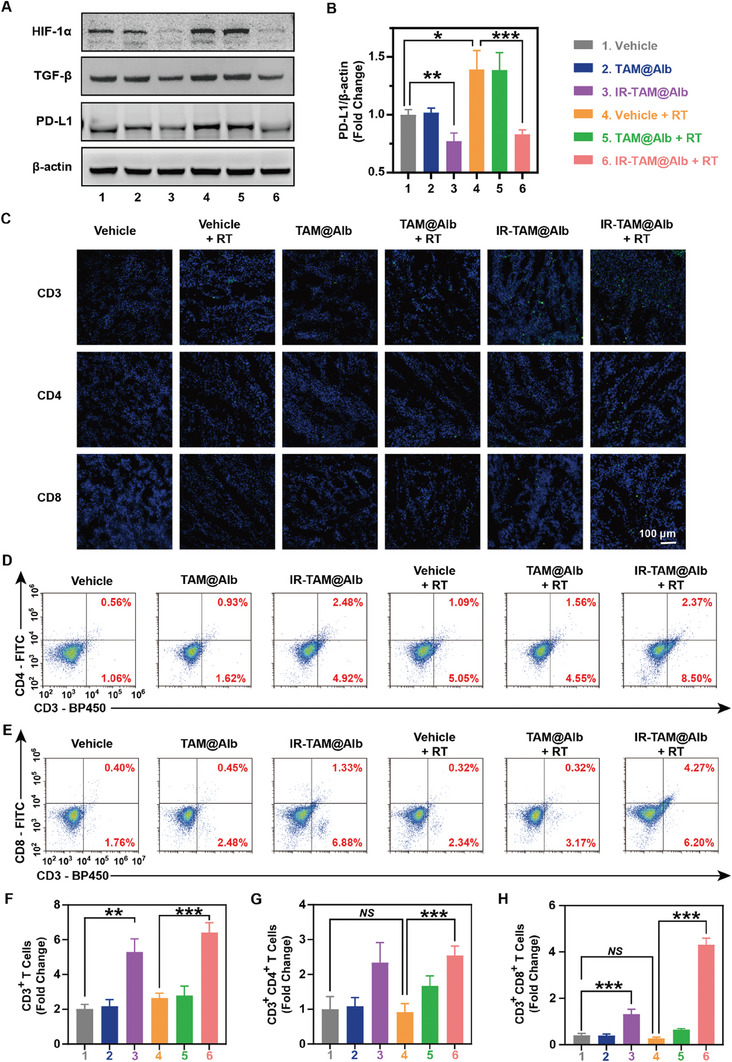
IR‐TAM@Alb nanoparticles downregulated the expression of PD‐L1 and TGF‐β protein to augment T cell infiltration, leading to the following reversed RT‐induced immunosuppression in vivo. A,B) Detection of PD‐L1, TGF‐β, and HIF‐1α protein expression by western blotting assay in mice bearing 4T1 tumors that received various treatments. The quantitative analysis of the expression of TGF‐β and PD‐L1 proteins was then performed by ImageJ (*n* = 3). C) The representative immunofluorescence images of CD3^+^, CD4^+^, and CD8^+^ T cells in 4T1 tumors following various treatments are presented (scale bar = 100 µm). D–H) Quantification of CD3^+^, CD4^+^, and CD8^+^ T cells in MB49 tumors after receiving different treatments by flow cytometry measurements. Data were demonstrated as mean ± SD. * *p* < 0.05, ** *p* < 0.01, and *** *p* < 0.001. *NS* means no significant difference, compared with the vehicle group.

Encouraged by the excellent in vitro PD‐L1 and TGF‐β down‐regulation ability of IR‐TAM@Alb, it was reasonable to speculate that this function can also be observed in vivo. To prove this, 4T1‐bearing mice were used (**Figure** **4**). As results demonstrated, IR‐TAM@Alb nanoparticles effectively suppressed the expression of PD‐L1 and TGF‐β proteins in tumors, as well as inhibiting tumor cell proliferation (Figure 4A,B; Figures S21 and S22, Supporting Information). This effect was observed despite RT's potential to induce PD‐L1 and TGF‐β expression (Figure 4A,B). It was widely acknowledged that the downregulation of PD‐L1 and TGF‐β1 simultaneously can most effectively ameliorate T cell exhaustion.^[^ ^4^, ^13^ ^]^ The other portion of the tumor tissue from each group was utilized for immunofluorescence analysis to observe the infiltration of T lymphocyte populations within the TME (Figure 4C). As a result of potent modulation of PD‐L1 and TGF‐β by IR‐TAM@Alb, either alone or in combination with RT, IR‐TAM@Alb significantly increased the infiltration of CD3^+^, CD4^+^, and CD8^+^ T cells into 4T1 tumors (Figure 4C). To further validate the immune‐enhancing effect of IR‐TAM@Alb in vivo, we also employed flow cytometry to quantitatively analyze T cell infiltration within tumors of MB49‐bearing mice with different treatments (Figure 4D–H). These findings demonstrated that IR‐TAM@Alb could effectively augment the infiltration of CD3^+^ and CD4^+^ T cells, regardless of combination treatment with RT or not (Figure 4D–H). In terms of CD8^+^ T cells, RT and IR‐TAM@Alb combination therapy exhibited a synergistic enhancement effect (Figure 4D–H). All of these findings demonstrated that IR‐TAM@Alb nanoparticles exhibited potent positive immunomodulatory efficacy in vivo via suppressing PD‐L1 and TGF‐β expression to prevent the possible acquired immune tolerance induced by RT.

### 2.8 The Combination Therapy of IR‐TAM@Alb Nanoparticles and RT Significantly Inhibited Local Tumor Growth and Remiss Tumor Metastatic In Vivo

**Figure 5:**
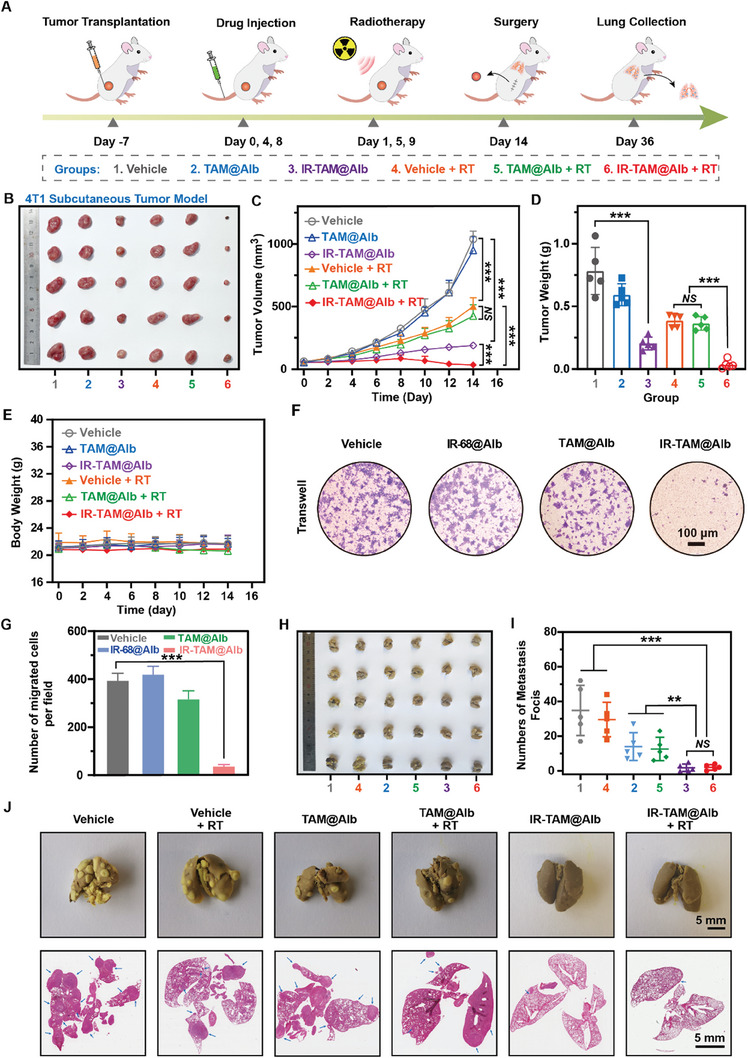
IR‐TAM@Alb nanoparticles mediated synergistic RT exhibited ideal anti‐tumor and anti‐metastasis effects. A) Schematic diagram of 4T1 tumor treatment process. B) The photograph of the 4T1 tumors on Day 14 after various treatments: 1. Vehicle; 2. TAM@Alb; 3. IR‐TAM@Alb; 4. Vehicle + RT; 5. TAM@Alb + RT; 6. IR‐TAM@Alb + RT (*n* = 5). C) The tumor growth curve of 4T1 tumors following various treatments (*n* = 5). D) Tumor weight of collected 4T1 tumors receiving different treatments at Day 14 (*n* = 5). E) The body weight changes of 4T1 tumor‐bearing mice in the period from the start of treatment to surgery (*n* = 5). F,G) Evaluation of the migration of 4T1 cells by the in vitro trans‐well migration assay after Vehicle, TAM@Alb, IR‐68@Alb, or IR‐TAM@Alb treatment (*n* = 3). H) The photograph of collected lungs to show 4T1 tumor metastasis after various treatments (*n* = 5). I) Quantification of the numbers of 4T1 metastasis focis in lungs after various treatments (*n* = 5). J) Representative morphology photographs of lung metastases and H&E staining from each group (Scale bar = 5 mm). Data were demonstrated as mean ± SD. ** *p* < 0.01, and *** *p* < 0.001. *NS* means no significant difference, compared with the vehicle group.

Inspired by the remarkable in vitro anticancer activity of IR‐TAM@Alb and its exceptional immune reactivation capacity in vivo, the 4T1 tumor model was then established to further evaluate the synergistic therapeutic effect of IR‐TAM@Alb and RT (**Figure** **5**). The therapeutic schedule was exhibited as Figure 5A, the maximal inhibition of 4T1 tumor growth was observed in the IR‐TAM@Alb + RT group post 14 Days treatment (Figure 5B–D). Moreover, the treatment with IR‐TAM@Alb alone also showed considerable tumor suppression (Figure 5B–D). TAM@Alb did not seem to gain such effect in treatment with or without RT (Figure 5B–D). The tumor volume growth curve and the final collected tumor mass both provided evidence of this phenomenon just mentioned above (Figure 5B–D). Furthermore, administration of IR‐TAM@Alb nanoparticles did not result in a significant alteration in the body weight of MB49 tumor‐bearing mice when compared to the Vehicle group, suggesting that IR‐TAM@Alb exhibited desirable biocompatibility in vivo (Figure 5E).

Inspired by the demonstrated efficacy of IR‐TAM@Alb in reversing amplified TGF‐β protein secretion following RT, we speculated that IR‐TAM@Alb may inhibit tumor metastasis both in vitro and in vivo (Figure 3). As results indicated, IR‐TAM@Alb exhibited the ability to impede cancer cell invasion in both 4T1 cells and MCF‐7 cells in vitro by transwell migration assay through the upregulation of E‐cadherin and down‐regulation of Vimentin (Figure 5F,G; Figures S23 and S24, Supporting Information). After the 4T1 tumors were surgically removed and the incision was well sutured, mice were continued to be fed without any treatment for 22 days to mimic postoperative lung metastasis (Figure 5A). As we expected, whether used in combination with or without RT, IR‐TAM@Alb demonstrated an excellent inhibitory effect on lung metastasis (Figure 5G–J). Compared to the Vehicle group, both the IR‐TAM@Alb group and the IR‐TAM@Alb group + RT group exhibited a significant reduction in the number of metastatic nodules in the lung (Figure 5H–J). In addition, TAM@Alb played a certain role in anti‐tumor metastasis (Figure 5H–J). However, RT alone had shown no efficacy against tumor metastases (Figure 5H–J). These results altogether suggested that IR‐TAM@Alb can synergize with RT to better suppress local tumor growth and concomitantly exert an inhibitory effect on lung metastasis.

To further evaluate the role of CD8 T cells in the anti‐tumor efficacy of the combination therapy of IR‐TAM@Alb and RT in local solid tumors, the breast fat pad implanted local 4T1 tumors were also used. As shown in Figure S25 (Supporting Information), after the treatments of IR‐TAM@Alb, the expression of HIF‐1α mRNA and HIF‐1α protein was decreased, meaning that the hypoxia tumor microenvironment may be remissed by IR‐TAM@Alb treatment. When co‐treated IR‐TAM@Alb with RT rather than IR‐TAM@Alb or RT treatment alone, the infiltration of NK cells was obviously increased while depressing the infiltration of MDSC cells in 4T1 tumors (Figures S26–S28, Supporting Information). Besides, after the usage of anti‐CD8 antibody to deplete CD8 T cells in 4T1 tumors, the tumor growth of 4T1 tumors was increased by IR‐TAM@Alb, RT, or co‐treated of IR‐TAM@Alb and RT when compared with the mice treated without CD8 depleting antibody (Figures S29 and S30, Supporting Information). Moreover, the tumor weight of IR‐TAM@Alb treated together with anti‐CD8 antibody was much larger than the mice treated with IR‐TAM@Alb alone (Figure S31, Supporting Information). Moreover, the proteomic analysis of IR‐TAM@Alb‐treated mammary fat pad implanted 4T1 tumors collected on Day 14 was conducted. As results showed in Figure S32 (Supporting Information), the immune‐resistance status of 4T1 tumors was reversed to some extent with enhanced immunoglobulin complex expression and amplified complement activation. All in all, the anti‐tumor efficacy of IR‐TAM@Alb was at least partly due to its capacity in enhancing T‐cell infiltration in tumors.

### 2.9 IR‐TAM@Alb Synergistically Enhanced RT and Induced Potent Anti‐Tumor Immune Memory Effect

**Figure 6:**
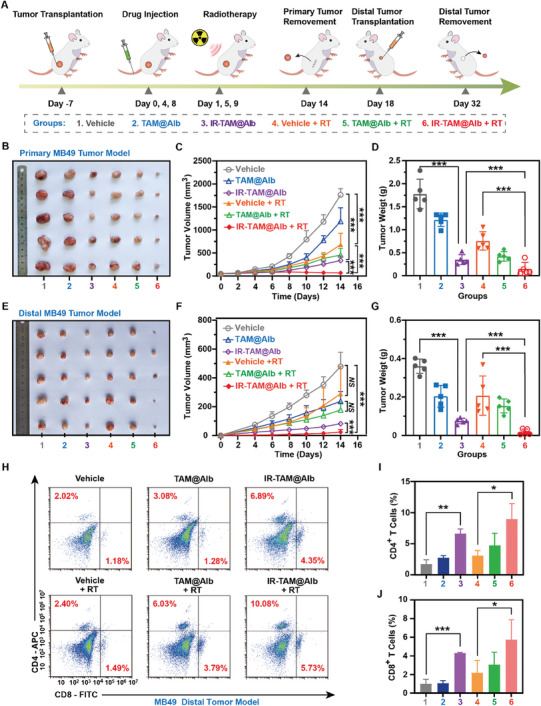
IR‐TAM@Alb synergized with RT to enhance the efficacy of antitumor immunological memory induction in MB49‐bearing mice. A) Schematic diagram of MB49 tumor treatment process. B) The photograph depicting the primary MB49 tumors collected on Day 14 (*n* = 5). C) Growth curves of the primary MB49 tumors (*n* = 5). D) Tumor weight of primary MB49 tumors collected on Day 14 (*n* = 5). E) Photograph of distal MB49 tumors collected from each group on Day 32 (*n* = 5). F) Growth curves of distal MB49 tumor volume (*n* = 5). G) Tumor weight of distal MB49 tumors collected on Day 32 (*n* = 5). H–J) Analysis of CD4^+^ T and CD8^+^ T cell populations in distal tumor tissues collected on Day 32 using flow cytometry (*n* = 5). Data were demonstrated as mean ± SD. * *p* < 0.05, ** *p* < 0.01, and *** *p* < 0.001. *NS* means no significant difference, compared with the vehicle group.

Considering the potential immune vaccine effect of RT reported in some studies, it was intriguing to investigate whether combining IR‐TAM@Alb with RT could further augment this effect.^[^ ^2^, ^4^ ^]^ Hence, MB49 tumor‐bearing mice were utilized to further validate the extensive anti‐tumor and distal anti‐tumor effects of the IR‐TAM@Alb nanoparticle combination with RT (**Figure** **6**A). In the local tumor model of MB49, the treatment effect of each group was similar to that of the 4T1 tumor model (Figures 5 and 6; Figure S33, Supporting Information). IR‐TAM@Alb combination therapy with RT demonstrated the most effective tumor suppression effect, with gradual reduction in the tumor volume observed during the latter half of treatment (Figure 6B–D). The final weight of local tumors was consistent with the growth curve of tumor volume (Figure 6B–D). Apart from this, the distal tumors of the IR‐TAM@Alb + RT group also exhibited a remarkably slow growth rate, indicating the excellent tumor immunosuppression (Figure 6E–G). Interestingly enough, IR‐TAM@Alb nanoparticles could already exhibit a potent immunogenic effect when administered alone (Figure 6E–G). Subsequently, abscopal MB49 tumors were collected for flow cytometry to evaluate the infiltration of T cells. As results showed, IR‐TAM@Alb combined with RT synergistically increased the infiltrating of CD3^+^, CD4^+,^ and CD8^+^ T cells within abscopal MB49 tumors (Figure 6H–J). All in all, these above findings suggested that the combination of IR‐TAM@Alb and RT elicited favorable tumor‐specific immune memory effects in treating both local and abscopal tumors.

### 2.10 IR‐TAM@Alb Selectively Accumulated in RIPF Area

Although RT technology was advancing toward precision, such as intensity‐modulated RT and conformal RT, it remained inevitable that some radiation would deviate from the target and result in associated complications, especially radiation‐induced pneumonia and RIPF.^[^ ^21^ ^]^ As we all know, with the development and deterioration of RIPF, the normal function of the lung was impaired, which then led to lung dysfunction and death of patients.^[^ ^4^, ^21^ ^]^ However, at present, no ideal strategy has been discovered to effectively prevent or cure RIPF.^[^ ^21^ ^]^ Very recent research revealed that IR780, a heptamethine cyanine dye, exhibited the ability to selectively accumulate in radiation‐affected lung tissue via OTAP targeting and may hold promise as a weak therapeutic agent for mitigating acute lung injury.^[^ ^21^ ^]^ Besides, it was also proved that some AMPK inducers could also relieve fibrosis‐related diseases like liver and lung fibrosis via TGF‐β inhibition to decrease the secretion of collagen, α‐SMA, and fibronectin.^[^ ^25^ ^]^ Thus, building upon these foundations, we believed that IR‐TAM@Alb composed of heptamethine cyanine dye IR‐68 and AMPK inducer TAM may possess RIPF‐targeting and highly effective treatment capacity.

To prove this, we conducted an investigation into the targeting capabilities of IR‐TAM@Alb at sites of RIPF at 8 weeks post‐RT (Figure S34A, Supporting Information).^[^ ^26^ ^]^ The representative morphology and H&E staining of the lungs are shown in Figure S34B (Supporting Information), which was consistent with the early stage of lung injury caused by RT, characterized by edema congestion and alveolar exudation. As we all know, activated fibroblasts were primarily responsible for the deposition of connective tissue during fibrosis, and researchers had demonstrated that heptamethine cyanine dye IR‐780 can be selectively internalized by fibroblasts through SLCO2A1 (one typical OATP).^[^ ^21^ ^]^ Theoretically, IR‐TAM@Alb may be preferentially taken up by the post‐radiation lung tissue by the same mechanism since IR‐TAM was also a heptamethine cyanine dye like IR‐780 (Figure S34C,D, Supporting Information). As shown in Figure S34C,D (Supporting Information), the expression of SLCO2A1 protein in lung tissue after RT was about twice that of the vehicle group. Then, in vivo fluorescence imaging was employed to assess the distribution of IR‐TAM@Alb within the lungs of mice that were either treated with or without RT (Figure S34E–G, Supporting Information). As results indicated, IR‐TAM@Alb selectively accumulated in the irradiated lung tissue, exhibiting fluorescence intensity more than 2‐fold stronger than the control group (Figure S34E–G, Supporting Information). Thus, IR‐TAM@Alb could selectively accumulated in RIPF.

### 2.11 IR‐TAM@Alb Effectively Suppressed RIPF and Exhibited the Potential for Diagnostic Application of RIPF

**Figure 7:**
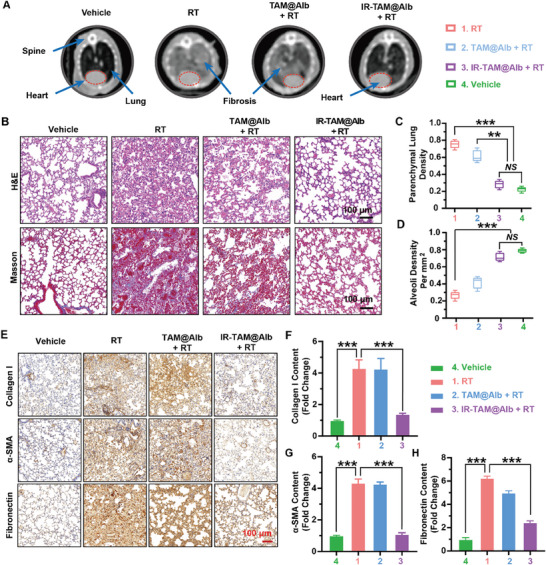
IR‐TAM@Alb nanoparticles effectively depressed the development of RIPF. A) The representative image of the Computed Tomography scans at week 16 post‐RT (15 Gy) from different treatments, the dashed red circle represents the location of the heart. B) The representative images of H&E staining and Masson staining of lungs after Vehicle, RT, TAM@Alb + RT, or IR‐TAM@Alb + RT treatment, scale bar = 100 µm. C,D) Quantification of the parenchymal lung density and alveolar density evaluated by H&E staining (*n* = 5). E) The typical immunohistochemical staining image of fibrosis‐related proteins in the lungs, including collagen I, α‐SMA, and fibronectin, scale bar = 100 µm. F–H) Quantitation analysis of fibrosis‐related proteins in the lungs, including collagen I, α‐SMA, and fibronectin (*n* = 5). Data were demonstrated as mean ± SD via a two‐tail Student's *t*‐test. ** *p* < 0.01, and *** *p* < 0.001. *NS* means no significant difference, compared with the vehicle group.

To further assess the therapeutic efficacy of IR‐TAM@Alb in mitigating RIPF, mice were subjected to 15 Gy of lung irradiation to establish typical RIPF models, followed by biweekly administration of drugs in different groups (Figure S35, Supporting Information). At week 16 post‐RT, the mice were observed and sampled (**Figure** **7**A). The clinical CT examination revealed significant parenchymal hyperdensity in the lungs of mice at 16 weeks after RT, indicating advanced‐stage severe fibrosis (Figure 7A; Figure S36, Supporting Information). While CT imaging of mice treated with IR‐TAM@Alb nanoparticles revealed a significant deceleration in the progression of pulmonary fibrosis, with no significant deviation in hounsfield unit values compared to the control mice (Figure S36, Supporting Information). Compared to the control group and IR‐TAM@Alb group, the weight of isolated lungs in the RT group and TAM@Alb group was significantly increased due to the severe pulmonary fibrosis accompanied by hemorrhage and edema (Figure S36, Supporting Information).

Then, we subsequently conducted a more comprehensive analysis of pulmonary tissue by H&E staining, masson staining, and detection of collagen I, α‐SMA, and fibronectin protein (Figure 7B–D). First, H&E staining was used to evaluate the characteristics of alveoli and lung parenchyma in different groups (Figure 7B). And, Masson's trichrome staining was utilized to quantify the collagen content in pulmonary tissue, which served as a crucial parameter for assessing pulmonary fibrosis (Figure 7B). These findings were in line with the CT assessment and gross lung specimen measurements that mice treated with IR‐TAM@Alb showed significant improvements in both alveolar normalization and inhibition of collagen production compared to controls (Figure 7C,D). Furthermore, immunohistochemistry was used to evaluate the expression of fibrosis‐related proteins in lung tissues (Figure 7E). Quantitation of collagen I, α‐SMA, and fibronectin proteins showed that RT could significantly up‐regulate fibrosis‐related proteins (Figure 7E–H). While the accumulation of these proteins only increased very slightly after IR‐TAM@Alb treatment, indicating that treatment with IR‐TAM@Alb significantly alleviated the progression of fibrosis by inhibiting structural tissue remodeling (Figure 7E–H). In addition, consistent with the results of CT images, the intensity of in vivo fluorescence imaging in different groups was proportional to the degree of pulmonary fibrosis (Figures S37 and S38, Supporting Information), which further verified the fibrosis targeting capacity of IR‐TAM@Alb. Taken together, IR‐TAM@Alb showed potential application value in the clinical diagnosis and treatment of radiation‐induced fibrosis.

## 3 Discussion

Currently, as a routine strategy for the palliative or curative treatments of solid tumors, RT is widely used in clinical in about 50% of cancer patients.^[^ ^6^, ^18^ ^]^ Apart from causing DNA damage to kill tumor cells, RT could also convert cold tumors to hot ones by upregulating antigen presentation, inducing immunogenic cell death, and enhancing leukocyte influx.^[^ ^2b^ ^]^ However, the efficacy of RT in re‐activating tumor immunotherapy was always limited due to the acquired upregulation of immune checkpoints like PD‐L1 and TGF‐β, which then resulted in the incredible undesired immune tolerance with characteristics like reduced leukocyte influx and impaired T cell function.^[^ ^4^, ^13^ ^]^ Besides, due to the impaired oxygen supply caused by the abnormal tumor vessels, the hypoxia status of tumor cells would limit the generation of ROS and DNA damage by RT.^[^ ^6^, ^16^ ^]^ Moreover, it was newly proved that apart from blocking T cell activity by PD‐1/PD‐L1 recognition, cytoplasm located‐PD‐L1 could speed up the DNA damage repair process by the stabilization of DDR‐related mRNAs.^[^ ^4^, ^7^ ^]^ Thus, the efficacy of RT was seriously limited due to the hostile TME, including tumor hypoxia, increased PD‐L1 expression, and acquired TGF‐β upregulation. In this study, to solve the obstacles of RT simultaneously and effectively, we newly revealed that apart from its usage as the adjuvant chemotherapeutic for the endocrine treatment of almost all stages of ER‐positive breast cancer, mitochondria dysfunction inducer TAM may could be used as a “one for all” PD‐L1/TGF‐β1 dual‐immune checkpoint inhibitor and hypoxia reversing agent to sensitize RT, which may extend the clinical application of TAM in the future (Figure 1). Then, to better decrease the dosage of TAM needed for RT sensitization and enhance its tumor‐targeting capacity, TAM was chemically conjugated with tumor‐targeting heptamethine cyanine dye IR‐68 to establish novel TAM derivate named IR‐TAM and then further self‐assembled with Alb to form IR‐TAM@Alb nanoparticles (Figures 1 and 2). Benefiting from this, IR‐TAM@Alb nanoparticles more effectively induced mitochondria dysfunction to reverse tumor hypoxia, inhibit TGF‐β secretion, and depress PD‐L1 expression at a relatively lower dosage of 4 µm in vitro, while the dosage was about 30 µm for free TAM or TAM@Alb group (Figure 3). Then, the combination therapy of IR‐TAM@Alb nanoparticles and RT rather than RT alone or IR‐TAM@Alb itself most effectively enhanced the generation amounts of DNA damage, amplified T cell infiltration, and enhanced T cell killing function in both bladder cancers and breast cancers, which finally almost totally inhibited local and absopal tumor growth, as well as preventing tumor metastasis (Figures 4, 5, 6). As we all know, apart from RT, some other tumor therapies may also face the acquired immune resistance of PD‐L1 and TGF‐β protein upregulation, including 5‐fluorouracil, and cisplatin‐based‐chemotherapy, adoptive cell therapy, oncolytic virus therapy, and bacteria therapy.^[^ ^14^, ^27^ ^]^ IR‐TAM@Alb may also be used as ideal adjuvant therapy to sensitize these tumor therapies via PD‐L1 and TGF‐β dual‐immune checkpoint inhibition (Figures 1, 2, 3, 4, 5, 6). All in all, the multifunctional IR‐TAM@Alb nanoparticles rather than the clinically used mono‐functional PD‐1, PD‐L1, or TGF‐β antibodies may be better used as an adjuvant to enhance the efficacy of tumor therapies in clinical in the future.

RIPF is a serious treatment complication that affects about 50% of cancer patients receiving RT for solid tumors.^[^ ^28^ ^]^ Generally, RIPF was a malignant irreversible disease that was characterized by progressive destruction of lung tissues, deterioration, or even loss of lung function.^[^ ^28^ ^]^ At last, as the disease progresses, RIPF can compromise the quality of human life and eventually lead to respiratory failure and death.^[^ ^28^ ^]^ As we all know, RT‐induced pneumonitis can induce the activation of fibroblasts/myofibroblasts to secret ECM proteins like collagen, α‐SMA, and fibronectin, which further resulted in pulmonary fibrosis.^[^ ^21^, ^28^ ^]^ But, unfortunately, the specific mechanisms by which RT causes RIPF had not been well revealed nor had effective and ideal treatments or targeted therapies for RIPF been developed or put forward.^[^ ^21^, ^28^ ^]^ As newly revealed by Chunmeng Shi et al., heptamethine cyanine dye like IR780 could selectively accumulate in the RIPF via the organic‐anion‐transporting polypeptide and slightly depress it.^[^ ^21^ ^]^ Besides, it was also proved that some AMPK inducers could also relieve fibrosis‐related diseases like liver and lung fibrosis via TGF‐β1 inhibition to decrease the secretion of collagen, α‐SMA, and fibronectin.^[^ ^25^ ^]^ Thus, we believed that IR‐TAM@Alb composed of heptamethine cyanine dye IR‐68 and AMPK inducer TAM may possess RIPF‐targeting and highly effective treatment capacity (Figure 7). As we proved in this study, IR‐TAM@Alb nanoparticles could also selectively accumulate in the area of RIPF to prevent or depress the radiation‐induced fibroblast activation by inhibiting collagen generation, α‐SMA deposition, and fibronectin production in Balb/C mice, which was also used for 4T1 tumor establishment (Figure 7). But, an equal dosage of TAM@Alb possessed no such capacity in preventing or curing RIPF. In the future, we will also use a more suitable RIPF evaluating model by using C57BL/6 mice to better evaluate the capacity of IR‐TAM@Alb in depressing RIPF, as well as using some other fibrosis models like hepatic fibrosis, pulmonary fibrosis, renal fibrosis, and et al., to evaluate the extensive feasibility of using IR‐TAM@Alb to depress different fibrosis diseases.^[^ ^29^ ^]^ All in all, in this well‐designed research, we designed a tumor/fibrosis dual‐targeting and co‐therapy strategy to eradicate RT‐resistant tumors while sparing normal lung tissues.

Recently, more overwhelming evidence proved that the activation of AMPK was also beneficial for the prevention, remission, and treatment of various chronic diseases including obesity, non‐alcoholic fatty liver disease, type 2 diabetes, cardiometabolic disease, and so on.^[^ ^17^, ^30^ ^]^ The development of new tactics for AMPK activators was expected to bring a breakthrough in the treatment of a variety of chronic diseases rather than only malignant tumors.^[^ ^18^, ^22^, ^30^ ^]^ Since TAM or IR‐TAM@Alb with AMPK activation was safe, cheap, and effective, IR‐TAM@Alb may also be used to prevent the occurrence or slow the development of these diseases. All in all, this simple but effective method using TAM or its derivate IR‐TAM@Alb could expand the clinical application field of TAM, further supporting its clinical translation, especially in tumor therapy sensitization, fibrosis prevention, type 2 diabetes treatment, as well as non‐alcoholic fatty liver disease therapy.

## 4 Conclusion

In this study, we newly proved that TAM, as the most widely used adjuvant chemotherapeutic for the endocrine treatment of almost all stages of ER‐positive breast cancer, could be used as a PD‐L1/TGF‐β dual‐immune checkpoint inhibitor, which may extend the clinical application of TAM in the future. To further solve the defects of TAM in lacking tumor‐targeting capacity, we chemically conjugated OXPHOS disruption anti‐tumor agent TAM with tumor/fibrosis dual‐targeting heptamethine cyanine dye IR‐68 to establish a novel TAM derivate named IR‐TAM and then further self‐assembled with Alb to form IR‐TAM@Alb nanoparticles. By doing this, IR‐TAM@Alb nanoparticles effectively reversed tumor hypoxia, depressed PD‐L1 expression, and inhibited TGF‐β secretion at a relatively lower dosage by inducing mitochondria dysfunction when compared with free TAM or TAM@Alb (TAM 30 µm versus IR‐TAM 4 µm). Then, IR‐TAM@Alb nanoparticles combination therapy with RT rather than RT alone or IR‐TAM@Alb nanoparticles itself most effectively converted cold tumors to hot ones via generation amounts of DNA damage and T cell infiltration in both bladder cancers and breast cancers to inhibit local and abscopal tumor growth, as well as preventing tumor metastasis. Apart from this, just as importantly, IR‐TAM@Alb nanoparticles also selectively accumulate in the area of RIPF, which then could prevent or depress the radiation‐induced fibroblast activation by inhibiting TGF‐β secretion, collagen generation, as well as fibronectin production. But, TAM@Alb possessed no such capacity to prevent or depress RIPF at the same dosage. So in short, in this well‐designed research, we designed a tumor/fibrosis dual‐targeting and co‐therapy strategy to eradicate RT‐resistant tumors while sparing normal lung tissues. This simple but effective method could expand the clinical application field of TAM and its’ derivate, especially in tumor therapy sensitization, fibrosis prevention, and treatment.

## Conflict of Interest

The authors declare no conflict of interest.

## Author Contributions

Z.Z., X.J., and L.Y. contributed equally to this work. Z.Z., Z.L., and J.S. conceived and designed the study. Z.Z., X.J., and L.Y. conducted the experiments and wrote the paper. Z.Z., X.J., L.Y., C.L., H.W., and W.X. analyzed and interpreted the data. Z.Z., Z.L., and J.S. supervised the research.

## Supporting information

Supporting Information

## Data Availability

The data that support the findings of this study are available in the supplementary material of this article.
