# Supplementary material for: Mitochondria Energy Metabolism Depression as Novel Adjuvant to Sensitize Radiotherapy and Inhibit Radiation Induced‐Pulmonary Fibrosis
Source: Adv Sci (Weinh). 2024 May 7;11(26):2401394. doi: 10.1002/advs.202401394 (PMC11234447; doi:10.1002/advs.202401394)
Supplement: Supplementary file 1 — Supporting Information [file ADVS-11-2401394-s001.docx]

**Mitochondria Energy Metabolism Depression as Novel Adjuvant to Sensitize Radiotherapy and Inhibit Radiation Induced-Pulmonary Fibrosis**

Zaigang Zhou ^a,#^, Xin Jiang ^c,#^, Lei Yi ^d,#^, Cheng Li ^e^, Haoxiang Wang ^a^, Wei Xiong ^e^, Zhipeng Li ^a,*^, Jianliang Shen^a,b,*^

^a^ National Engineering Research Center of Ophthalmology and Optometry, Eye Hospital, Wenzhou Medical University, Wenzhou 325027, China.

^b^ Zhejiang Engineering Research Center for Tissue Repair Materials, Wenzhou Institute, University of Chinese Academy of Sciences, Wenzhou, Zhejiang 325001, China.

^c^ Department of Urology, Xiangya Hospital, Central South University, Changsha, Hunan 410008, China.

^d^ Department of Urology, The Second Xiangya Hospital, Central South University, Changsha, Hunan 410011, China.

^e^ Department of Urology, The Third Xiangya Hospital, Central South University, Changsha, Hunan 410013, China

^#^ These authors contributed equally to this paper.

*** Corresponding authors:**

113686@wmu.edu.cn (Zhipeng Li), sjl1@wmu.edu.cn (Jinaliang Shen).

**1. Materials and methods**

**1.1 Materials**

Unless otherwise noted, all chemicals were bought from Shanghai Aladdin Reagent Co., Ltd. Bovine Serum Albumin (Alb) was obtained from Sigma Chemical Co. Ltd. Roswell Park Memorial Institute (RPMI) 1640 medium, Fetal bovine serum (FBS), and trypsin-Ethylenediaminetetraacetic acid disodium salt were purchased from GIBCO-BRL (Grand Island, NY, USA). All reagents of the western blotting assay were acquired from Beyotime Biotechnology (Shanghai, China). Rabbit anti-mouse AMPK and rabbit anti-mouse p-AMPK (Thr 172) were all obtained from Cell Signaling Technology (USA). Rabbit anti-mouse antibody or rabbit anti-human PD-L1 antibody, rabbit anti-mouse HIF-1α antibody, rabbit anti-mouse E-cadherin antibody, rabbit anti-mouse Vimentin antibody, rabbit anti-mouse Calreticulin (CRT) antibody, rabbit anti-mouse Ki67 antibody, and rabbit anti-β-actin antibody were purchased from Affinity Biosciences LTD (USA). DyLight 488 labeled HIF-1α monoclonal antibody was obtained from Thermal Fisher Inc. (USA). APC-conjugated anti-mouse PD-L1 antibody, BP450-conjugated anti-mouse CD3 antibody, FITC-conjugated anti-mouse CD4 antibody, FITC-conjugated anti-mouse CD8 antibody, PerCP-conjugated anti-mouse CD11c antibody, PerCP-conjugated anti-mouse CD11c antibody, FITC-conjugated anti-mouse CD49b antibody, and Brilliant Violet 421^™^-conjugated anti-mouse CD45 antibody were purchased from BioLegend, Inc. (USA). Anti-CD8 antibody for *in vivo* CD8 T cell depleting was obtained from BioXcell company. All antibodies were diluted according to the manufacturer's instructions. Dorsomorphin (Com C) and recombinant mouse interferon-γ (IFN-γ) were provided by MedChemExpress (USA) and Pepro Tech Inc. (USA), respectively.

**1.2 Synthesis of IR-68**

IR-68 was prepared as previously reported.[1] Generally, N-butyl-2,3,3-trimethylindoleninium iodide (2.0 g, 5.9 mmol), N-[(3-(anilinomethylene)-2-chloro-1-cyclohexen-1-yl)-methylene]aniline monohydrochloride (6.5 mmol, 2.4 g), sodium acetate (5.9 mmol, 480 mg), and 1-(5-carboxypentyl)-2,3,3-trimethylindoleninium bromide (5.9 mmol, 2.4 g) in 75 mL of anhydrous ethanol at room temperature. The reaction mixture was heated to reflux at 80 ℃ in an oil bath for 5 h, after which the reaction was stopped. IR-68 was further purified by high preparative performance liquid chromatography (31.6%, 1.40 g, ∼94% pure). HR-MS (ESI-TOF) calculated for C_40_H_50_ClN_2_O_2_ [M]^+^ 625.355, found 625.355.

**1.3 Synthesis of IR-TAM**

TAM-NH was prepared as previously reported.[2] Generally, TAM (1.5 g, 4.1 mmol) was dissolved in 60 mL anhydrous toluene (6 mL) at 0 ℃, followed by the addition of superfluous chloroethyl chloroformate. After 30 min, the reaction was heated to reflux and kept for 48 h under inert environments. The reaction mixture was then concentrated in a vacuum to obtain a solid. Then, 60 mL methanol was added and the reaction solution was again heated at reflux for another 3 h. Finally, TAM-NH was purified by high-preparative performance liquid chromatography high-performance preparation liquid.

To prepare IR-TAM, TAM-NH, and IR-68-NHS were added to anhydrous dichloromethane. 48 h later, IR-TAM was purified by high preparative performance liquid chromatography (48.2%, ∼95% pure). Characterization data for IR-TAM: ^1^H NMR (400 MHz, DMSO-d_6_, ppm): 0.83 (t, 3 H), 0.93 (t, 3 H), 1.51 (b, 4 H), 1.59 (br., 2 H), 1.63 - 1.66 (s, 12 H), 1.70 (br., 4 H), 2.17 - 2.47 (m, 4 H), 2.68 (d, 4 H), 2.77 - 2.95 (m, 3 H), 3.49 - 3.59 (m, 2 H), 3.84 - 3.96 (m, 2 H), 4.20 (m, 4 H), 5.57 (d, 2 H), 6.31 (d, 2 H), 6.54 (dd, 2 H), 6.70 (t, 2 H), 7.06 - 7.12 (m, 3 H), 7.14 - 7.19 (m, 4 H), 7.24 -7.30 (m, 3 H), 7.32 - 7.38 (m, 2 H), 7.38 - 7.47 (m, 4 H), 7.62 (t, 2 H), 8.20 - 8.29 (m, 2 H). HR-MS (ESI-TOF) calculated for C_65_H_75_ClN_3_O_2_ [M]^+^ 964.554, found 964.556.

**1.4 Preparation and characterization of** **self-assembly IR-TAM@Alb nanoparticles**

IR-68@Alb and IR-TAM@Alb were prepared according to the previously reported strategy.[1, 3] Generally, IR-TAM (4 mg) was dissolved in 0.5 mL of dimethyl sulfoxide (DMSO), followed by the addition of 200 mg Alb dissolved in 40 mL ultrapure water with agitation. The dissolved IR-68 or IR-TAM was then added dropwise to the Alb solution and stirred for another 2 h, after which the excess water and DMSO were subsequently filtered off using an ultrafiltration cup with a membrane size of 30 kDa to obtain a concentrated dye-protein complex. To obtain stable nanoparticles with uniform diameters, the concentrated complexes were heated in a water bath at 70 ℃ for 10 minutes and subsequently cooled to room temperature, ultimately resulting in the preparation of nanoparticles. TAM@Alb was prepared by adopting similar methods reported previously.[1, 3c]

The mean particle size was measured using dynamic light scattering (DLS, Zetasizer Nano ZS ZEN3600). Transmission electron microscopy (TEM, FEI Talos) was performed to confirm the morphology of IR-TAM@Alb self-assembly nanoparticles. Moreover, the UV-Vis-NIR spectrometer (CARY5000, Agilent, America) and the NIR fluorescence spectrometer (Horiba, FluoroMax-4) were employed to evaluate the optical features of previously mentioned compounds and nano-systems.

**1.5 Cell culture and animal model establishing**

The MB49 (mouse bladder cancer cells), 4T1 (mouse breast cancer cells), 5637 (human bladder cancer cell), MCF-7 cells, and MDA-MB-231 (human breast cancer cells) cell lines, derived from the American Type Culture Collection, were cultured in RPMI-1640 medium (Gibco) supplemented with 10% fetal bovine serum (FBS) and 1% antibiotics (penicillin and streptomycin) at 37°C under a humidified atmosphere of 5% CO_2_.

The Laboratory Animal Center of Zhejiang Province (Hangzhou, China) provided female C57BL/6 mice and BALB/C mice (8 weeks old, ~ 20 g). Ectopic subcutaneous tumor models were established by subcutaneously injecting 4T1 or MB49 cells into the lower abdomen or armpit of mice. The mice were sacrificed at appropriate time points according to the experimental design, and tumors or metastases were collected for further study. All animal experiments were conducted by the guidelines of Wenzhou Institute, University of Chinese Academy of Sciences (Issue NO.): WIUCA 22021404) and under the protocols established by the National Regulation of China for Care and Use of Laboratory Animals.

**1.6 Molecular docking experiment**

Firstly, the Alb protein structure (PDB ID: 1N5U) was retrieved from the Protein Data Bank (PDB) database and processed using Pymol2.3.0 to eliminate crystal water and the original ligand, among other factors. Subsequently, AutoDocktools (v1.5.6) was employed for hydrogenation, charge calculation, charge assignment, and atom type designation. Next, we utilized ChemBioDraw Ultra 14.0 to depict the structures of small molecules and then imported them into ChemBio3D Ultra 14.0 for energy minimization. Finally, the interaction mode of the docking results was analyzed using PyMOL2.3.0 and Ligplot V2.2.5 with adjusted parameters.

**1.7 Protein binding assay**

Warfarin, ibuprofen, digoxin, and quinidine exhibit high affinity for albumin binding sites I, II, III, and α1-glycolipoprotein respectively. Therefore, following preincubation with 5% Alb at room temperature for 30 minutes and subsequent incubation with 2 μM IR-TAM for another 30 minutes, the fluorescence intensity was measured to investigate the binding site of IR-TAM with Alb.

The binding of Alb to IR-TAM was also observed using sodium dodecyl sulfate-polyacrylamide gel electrophoresis (SDS-PAGE) combined with NIR fluorescence. The resulting dye-protein complexes were stained with Coomassie Brilliant Blue and imaged using an in vivo imaging system (IVIS Lumina XRMS Series III). Additionally, fluorescence spectroscopy and UV absorption spectrum were performed on IR-TAM (final concentration: 2 μM) dissolved in varying concentrations of albumin.

**1.8 Mitochondrial complexes activity assay**

The respective concentration gradients of TAM and IR-TAM were prepared with PBS, and then their effects on mitochondrial function were evaluated by reaction with MitoCheck^®^ Complex Ι Activity Assay Kit, MitoCheck^®^ Complex Ⅱ Activity Assay Kit, and MitoCheck^®^ Complex Ⅳ Activity Assay Kit (Cayman, USA).

**1.9 Detection of ADP / ATP ratio change**

1 × 10^4^ 4T1 cells were cultured in confocal dishes overnight and subsequently treated with IR-TAM@Alb at varying concentrations or different groups containing Vehicle, TAM@Alb, IR-68@Alb, or IR-TAM@Alb (calculated by TAM, IR-68, or IR-TAM concentration: 4 μM) for 12 h. The cells were then harvested and lysed for the ADP/ATP Ratio Kit (Vigorous Biotechnology, China) under the instructions provided by the manufacturer.

**1.10** **[Subcellular localization](javascript:;) of IR-TAM@Alb nanoparticles**

After 4T1 cells were treated with IR-68@Alb (final concentration: 2 μM) or IR-TAM@Alb (final concentration: 2 μM) for 4 h, the cells were subjected to PBS washing followed by Mito Tracker Blue staining for 20 min. At last, CLSM (IVIS Lumina XRMS Series III) was employed for fluorescence image collection.

**1.11 Cellular uptake mechanism of IR-TAM@Alb nanoparticles**

To investigate the cellular uptake mechanism, 1.5 × 10^5^ 4T1 cells were cultured in confocal dishes for 24 h. Subsequently, the cells were pre-treated with chlorpromazine (CPZ, 20 μM), sulfobromophthalein (BSP, 250 μM), or kept at 4 ℃ for 1 h respectively. The cells were then incubated with IR-TAM@Alb nanoparticles at a concentration of 2 μM for an additional 4 h before being evaluated by CLSM and flow cytometry assay.

**1.12 Cell viability assay and clone formation assay**

Cell Counting Kit-8 (CCK-8) assay was used to measure the cell viability of MB49 and 4T1 cells under various conditions. The cells were cultured in RPMI-1640 medium containing 10% FBS and seeded in 96-well culture plates, maintained at a typical culture environment of 37℃ with 5% CO2. Following adhesion to the wall, tumor cells were treated with vehicle, TAM@Alb, IR-68@Alb, or IR-TAM@Alb nanoparticles at different concentrations for 24 h. Subsequently, tumor cells were subjected to RT or not, and cultured for an additional 48 h in replacement of the normal medium. At last, the tumor cells were incubated with CCK-8 reagent for 2 h, followed by detection of absorbance at a wavelength of 450 nm using a microplate reader (BIOTEK, Vermont, USA).

The clone formation assay was performed to further assess the proliferative capacity of the cell. Therefore, MB49 cells were first incubated with vehicle, TAM@Alb (2 μM), IR-68@Alb (2 μM), and IR-TAM@Alb (2 μM) for 24 h, and followed by RT (3 Gy, Efficiency: 6 Gy/min), all of the treatments were cleared and MB49 cells were cultured for 48 h sequentially. Then, 1×10^3^ cells were acquired from each group and seeded into 6-well plates evenly. After cultivation for 10 days, the image of clones was collected and crystal violet was finally applied to display the clones.

**1.13 Western blot and Real-time quantitative PCR (RT-qPCR) assay**

Following even seeding and 24-hour incubation in culturing dishes, cells were treated with TAM@Alb, IR-68@Alb, IR-TAM@Alb, or blank medium as a vehicle for an additional 24 h. Subsequently, the cells were collected and lysed using RIPA buffer supplemented with EDTA, phosphatase inhibitors, phenylmethanesulfonyl fluoride, and protease in appropriate proportions for 20 minutes. The lysates were then centrifuged at 12,000 rpm and at 4℃ for 20 minutes. Next, the Bicinchoninic acid (BCA) protein assay was conducted to determine the total protein concentrations of each group. Subsequently, equivalent amounts of prepared proteins were separated by SDS-polyacrylamide gel electrophoresis and transferred onto PVDF membranes. After blocking with 5% Alb for 1 hour, the corresponding primary antibodies and secondary antibodies were incubated with the PVDF membranes for the indicated time. Protein bands were detected using the ECL reagent and Imaging System (Peiqing Science and Technology) for visualization.[4]

Total RNA was extracted from cells using TRIzol reagent (Invitrogen, Carlsbad, CA, USA) following the manufacturer's protocol. Subsequently, reverse transcription of total RNA was performed with a reverse transcription kit (#RR047A, Takara, Japan). Real-time qPCR analysis was conducted to evaluate mRNA expression levels utilizing a PCR kit (#RR820A, Takara, Japan).[5]

**1.14 T cell-mediated tumor cell killing assay *in vitro***

Human blood was collected from a healthy volunteer following the approved protocol of the Ethics Committee at Xiangya Hospital of Central South University. All procedures were conducted in compliance with institutional ethical standards. In brief, peripheral blood mononuclear (PMBC) cells were obtained from whole blood using a Lymphocyte Separation Tube. T cells were subsequently isolated from the PMBC population utilizing EasyStep™ Human T Cell Isolation Cocktail (50 μL/mL, Stemcell), EasyStep™ Dextran RapidSpheres™ (40 μL/mL, Stemcell), and an isolation magnet (Stemcell). The isolated T cells were cultured in ImmunoCult™-XF T Cell Expansion Medium and activated with ImmunoCult™ Human CD3/CD28/CD2 T Cell Activator (Stemcell). Then, activated T cells were co-cultured with drug-pretreated 5637 cells or MDA-MB-231 cells (at a ratio of 10:1) and supplemented with anti-CD3 antibody (100 ng/mL, eBioscience, Thermo Scientific) and IL-2 (10 ng/mL). After another 24 h, the T cells and the medium of each group were collected for RT-qPCR, while the survival tumor cells were fixed and stained by crystal violet.

**1.15 Immunofluorescence assay** ***in vitro***

Cells were fixed with 4% paraformaldehyde at room temperature for 15 minutes after being subjected to different treatments according to grouping. If the target proteins are located in the cytoplasm and nucleus, cells were permeabilized in 0.5% Triton X-100 for 10 minutes and blocked by 5% bovine serum albumin for one hour. Subsequently, samples of cells were incubated with related primary antibodies overnight at 4 ℃, followed by fluorescent secondary antibodies for one hour and DAPI staining for 10 minutes at room temperature to visualize the target proteins and nucleus. Finally, the samples were observed under a fluorescence microscope.

**1.16 *In vivo* fluorescence imaging**

To assess the effect of IR-TAM@Alb on T-cell infiltration and associated antibody expression of tumors *in vivo*, mice were euthanized following various treatments to harvest tumor samples for subsequent Immunofluorescence analysis. Tumor tissue was embedded in OCT blocks and cut into 7 μm tumor tissue sections using a freezing microtome. After the removal of OCT with acetone, sections were permeabilized with 0.1% Triton X-100, blocked with 5% BSA, and incubated overnight at 4 °C with various primary antibodies, and then incubated with fluorescent secondary antibodies at the indicated concentrations, followed by DAPI staining. The use of PBS for cleaning is mandatory between successive steps. Finally, fluorescence images were acquired using CLSM.

**1.17 Biosafety evaluation of IR-TAM@Alb nanoparticles**

The mice were administered with IR-TAM@Alb (calculated by IR-TAM concentration: 10 mg/kg) via tail vein injection, as previously described. Based on the time of sacrifice, the mice were divided into acute or sub-acute groups at 48 hours or 2 weeks post-injection, respectively. Major organs were subsequently harvested for hematoxylin and eosin (H&E) staining to evaluate structural damage. Additionally, blood collected from the orbit of mice was used to assess potential toxic side effects on hepatic and renal function by detecting the levels of aspartate aminotransferase (AST), alanine aminotransferase (ALT), creatinine (CR), and blood [urea](javascript:;) [nitrogen](javascript:;) (BUN).

**1.18 Hemolysis assay**

# Firstly, fresh blood derived from the orbits of C57BL/6 mice was blended with sodium citrate for anticoagulation. Next, the mixture was centrifuged at 2000 rpm for 4 minutes to extract the red blood cells (RBCs). After that, a series of concentrations of IR-TAM@Alb nanoparticles (calculated by the IR-TAM concentration) prepared were mixed with the RBCs suspension to evaluate the hemolysis characteristic, and adding physiological saline for negative control while distilled water for positive control. After incubation at 37°C for 2 h, the samples were centrifuged to obtain their corresponding supernatants. Finally, the hemolysis ratio was determined by measuring the ultraviolet absorption of the supernatants at 541 nm.

**1.19 Evaluation of the anti-tumor efficacy of IR-TAM@Alb nanoparticles in the subcutaneous or orthotopic breast fat pad-implanted 4T1 tumor model**

5×10^5^ 4T1 cells were subcutaneously inoculated in the lower right flank region of the female BALB/C mouse to establish the 4T1 subcutaneous tumor model. After different treatments (Groups: Vehicle, 5 mg/kg TAM@Alb, 5 mg/kg IR-68@Alb, and 5 mg/kg IR-TAM@Alb) on Day 0, 4, 8, the mice were subjected to local radiation (3 Gy, 6 Gy/min) in the tumor area 24 h later. The tumor volume and body weight of the mice were measured every 2 days for two weeks. The tumor volume was calculated using the following formula: Tumor Volume = 0.5 × width^2^ × length. On Day 14, once the mice were under anesthesia, the primary tumor was meticulously excised and the surgical site was delicately closed using sutures to ensure that the tumor was removed as clean as possible and mice avoided postoperative infection. The mice were then fed with a standard diet until Day 36, and then their lung tissues were harvested for subsequent analysis.

5×10^5^ 4T1 cells were injected into the breast fat pad near the right abdomen of the female BALB/C mouse to establish the local 4T1 tumor model. When the tumor volume was about 50 mm^3^, the mice were randomly divided into eight groups: 1. Vehicle; 2. RT; 3. IR-TAM@Alb; 4. IR-TAM@Alb + RT; 5. anti-CD8 antibody; 6. RT + anti-CD8 antibody; 7. IR-TAM@Alb + anti-CD8 antibody; 8. IR-TAM@Alb + RT + anti-CD8 antibody (calculated by IR-TAM concentration: 5 mg/kg; anti-CD8 antibody dosage: 10 mg/kg). Different treatments like IR-TAM@Alb or anti-CD8 antibody were given on Days 0, 4, and 8, the mice were subjected to local radiation (3 Gy, 6 Gy/min) in the tumor area 24 h later. The tumor volume and body weight of the mice were measured every 2 days for two weeks. The tumor volume was calculated using the following formula: Tumor Volume = 0.5 × width^2^ × length. On Day 14, the mice were sacrificed to collect 4T1 tumors, followed by CD3^-^CD49b^+^natural killer (NK) cells detection and CD45^+^Gr-1^+^CD11b^+^ myeloid-derived suppressor cells (MDSC) detection through flow cytometry assay, as well as proteomic analysis.[5-6]

**1.20 The anti-tumor efficacy of IR-TAM@Alb nanoparticles in the MB49 model**

The female C57BL/6 mouse was subcutaneously inoculated with 5 × 10^5^ MB49 cells in the lower right flank region to establish the primary tumor. When the tumor reached a volume of approximately 50 mm^3^ (Day 7), the mice were grouped in a randomized manner (n = 5) and intravenously administered with Vehicle, 5 mg/kg TAM@Alb, or 5 mg/kg IR-TAM@Alb on Day 0, Day 4, and Day 8. Subsequently, the primary tumors were subjected to local radiotherapy or not (3 Gy, Efficiency: 6 Gy/min) at 24 hours post-injection of different drugs. The tumor volume and body weight of the mice were measured every 2 days for two weeks. The tumor volume was calculated based on the formula: Tumor Volume = 0.5 × width^2^ × length. After the mice were anesthetized, the primary tumor was excised and the surgical site was closed with sutures on Day 14. After a 4-day recovery period, the upper distal tumor (5 × 10^5^ MB49 cells) was transplanted under the axilla on the opposite side of each group’s mice. No further treatment was administered and the distal tumor was harvested on Day 32 for further analysis.

**1.21 IR-TAM@Alb nanoparticle for the diagnosis and treatment of radiation-induced pulmonary fibrosis (RIPF)**

Following anesthesia, BALB/C mice were exposed to a 15 Gy radiation dose targeting the pulmonary region to establish an animal model of radiation-induced lung injury. After 8 weeks, different groups of mice were administered IR-TAM@Alb via the tail vein. The fluorescence of mouse lungs was observed using an *in vivo* imaging system one day later, and lung tissue was subsequently extracted for fluorescence detection to assess the targeting efficacy of IR-TAM@Alb against radiation-induced lung injury.

As previously stated, BALB/C mice were exposed to radiation (15 Gy) targeting the pulmonary region to establish RIPF. The mice were allocated into four groups at random: Vehicle (without RT), RT, TAM@Alb + RT， IR-TAM@Alb + RT (Drug concentration: 2 mg/kg TAM, 2 mg/kg IR-TAM). The mice in the corresponding groups were intravenously injected with different drugs once every two weeks. At week 16, the mice underwent anesthesia and their lungs were imaged using CT with C-120 and W 1000 settings. Next, mice were sacrificed to collect their lungs for further analysis. The weight of the lung tissue was recorded, and the volume size of the lung tissue was measured using the drainage method. Then, the lung tissues were fixed with 4% paraformaldehyde for HE staining, Masson staining, and immunohistochemical staining of related proteins (Collagen I, α-SMA, and Fibronectin).

**1.22 Statistical analysis**

All results were presented by employing the methods of means ± standard deviation (S.D.). Statistical analysis was executed by adopting a two-tailed Student’s *t*-test for the comparison of two groups and a one-way ANOVA test for multiple groups. * *p* < 0.05 represents statistically significant; ** *p* < 0.01, and *** *p* < 0.001 stand for extremely significant; NS means no significant difference.


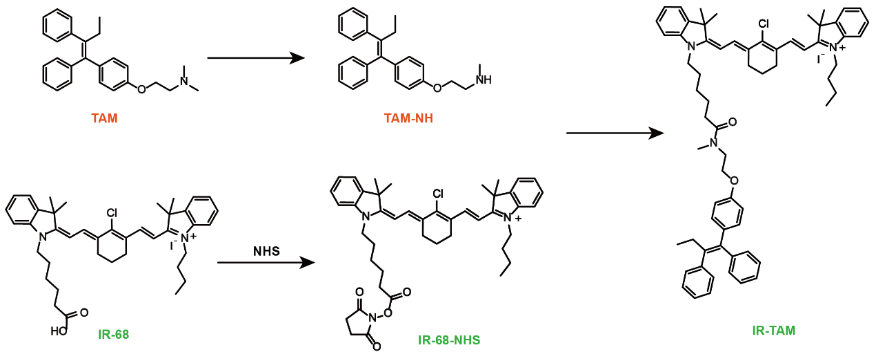


**Figure S1**. Synthetic route of IR-TAM.


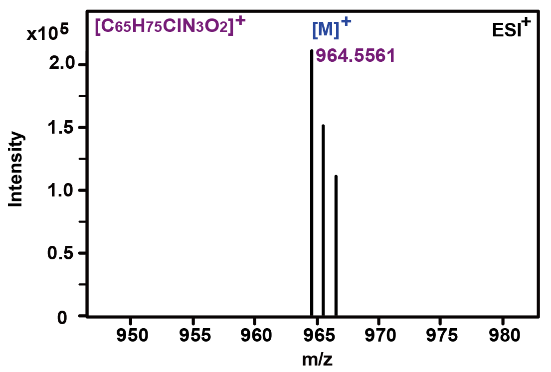


**Figure S2**. High-resolution mass spectrum (HR-MS) of IR-TAM.


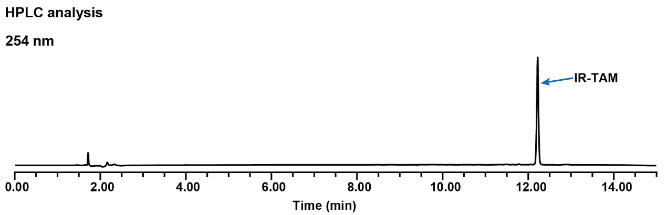


**Figure S3**. HPLC analysis to detect the purity of IR-TAM.


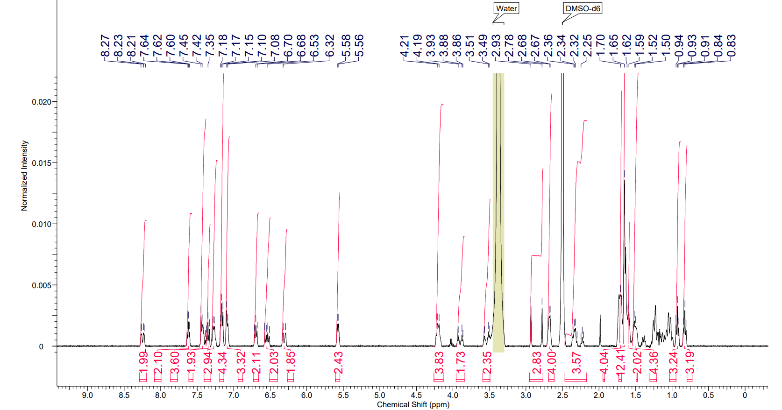


**Figure S4.** The ^1^H-NMR spectrum of IR-TAM


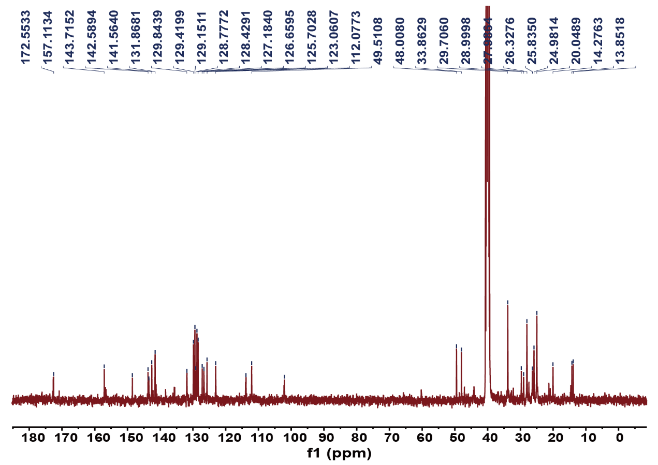


**Figure S5.** The ^13^C-NMR spectrum of IR-TAM


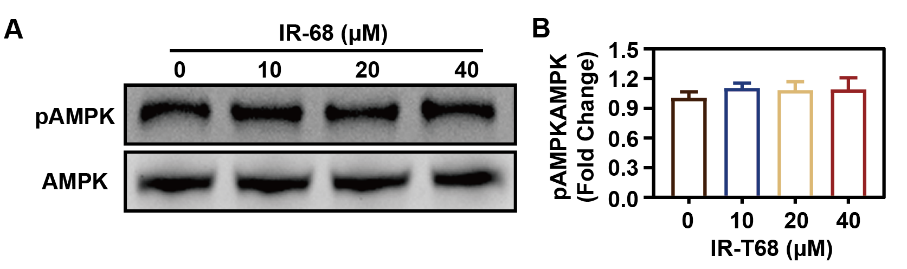


**Figure S6**. Detection of p-AMPK and AMPK protein expression in 4T1 cells by western blotting assay after treatment with different concentrations of IR-68 for 24 h and further quantified by ImageJ (n = 3). Data were demonstrated as mean ± SD.


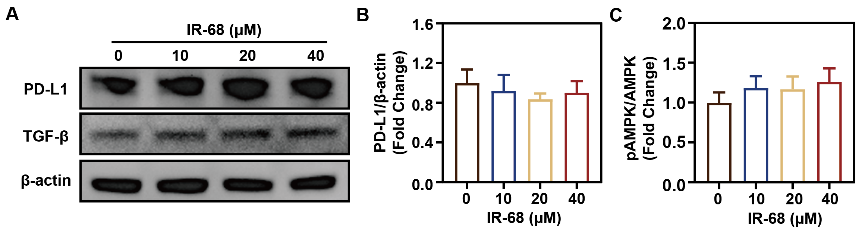


**Figure S7.** Detection of TGF-β1 and PD-L1 protein expression in 4T1 cells by western blotting assay after treatment with different concentrations of IR-68 for 24 h and further quantified by ImageJ (n = 3). Data were demonstrated as mean ± SD.


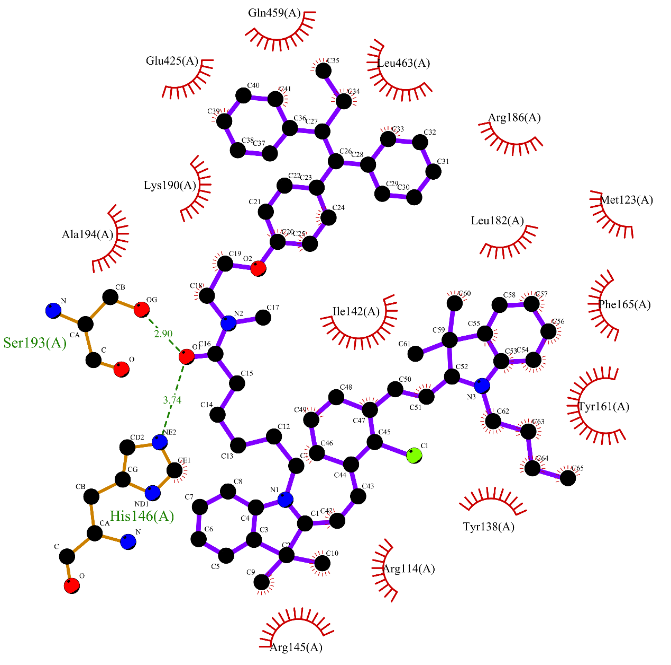


**Figure S8.** Molecular docking to reveal the binding site of Alb with IR-TAM and its high-affinity Alb-bond site, which mainly forms hydrogen bonds and hydrophobic forces between Alb and IR-TAM. IR-Tam formed hydrogen bonds with the Ser193 and His146 of Alb with lengths of 2.90 Å and 3.74 Å, respectively. IR-Tam formed hydrophobic forces with the Arg145, Arg114, Tyr138, Tyr161, Phe165, Met123, Leu182, Ile142, Arg186, Leu463, Gln459, Glu425, Lys190, and Ala194 of Alb.


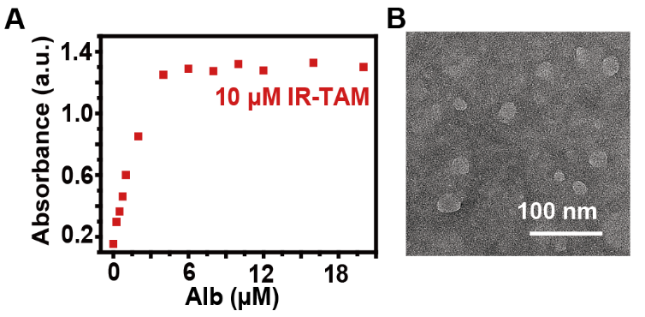


**Figure S9.** (A) The UV-Vis absorption spectra of IR-TAM in different concentrations of Alb. (B) TEM images of IR-TAM@Alb nanoparticles, scale bars = 100 nm.


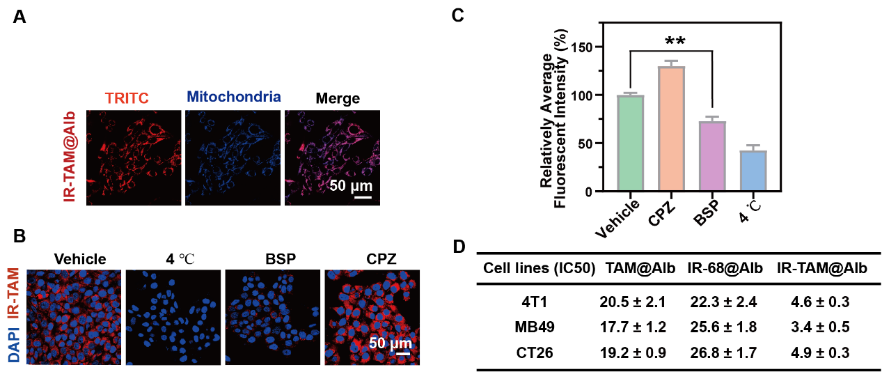


**Figure S10.** (A) Mito-Tracker Blue was applied to determine the co-location of mitochondria with IR-TAM@Alb. (B-C) CLSM was used to explore the uptake mechanism of IR-TAM@Alb via indicated pretreatments of 4 ℃, BSP, or chlorpromazine (CPZ) for 1 h, and the relatively average fluorescent intensity was quantified by ImageJ. (D) The IC50 of each cell line was calculated by PRISM. Data were demonstrated as mean ± SD. ** *p* < 0.01.


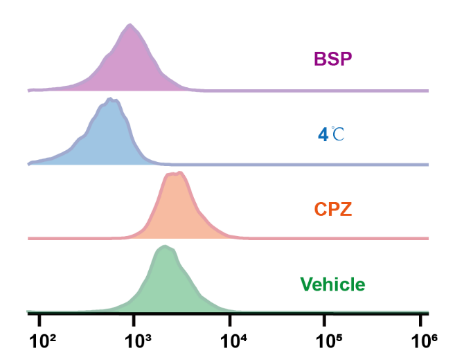


**Figure S11.** Flow cytometry assay to analyze the uptake mechanism of IR-TAM@Alb via the indicated pretreatment of 4 ℃, BSP, chlorpromazine (CPZ) for 1 h.


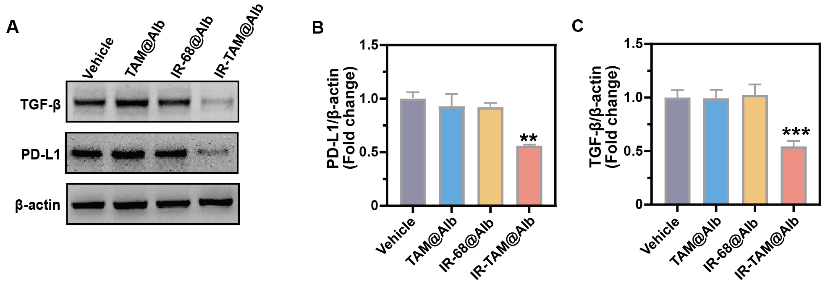


**Figure S12.** Detection of the expression levels of PD-L1 and TGF-β proteins in 4T1 cells treated with TAM@Alb, IR-68@Alb, or IR-TAM@Alb (calculated by TAM, IR-68, or IR-TAM concentration: 6 μM) by western blotting assay and then quantified by ImageJ, n = 3. Data were demonstrated as mean ± SD. ** *p* < 0.01, and *** *p* < 0.001.


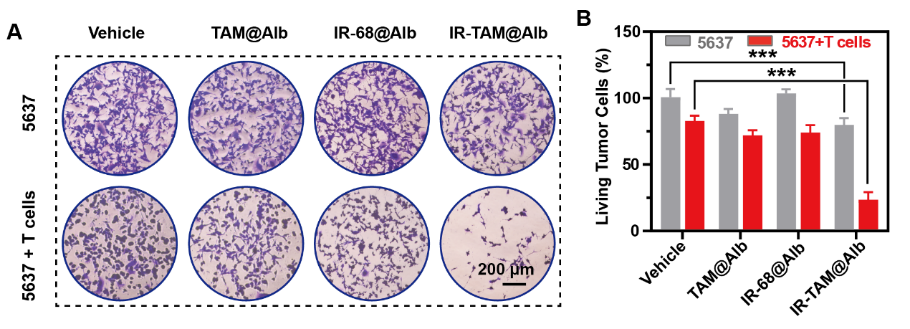


**Figure S13.** IR-TAM@Alb promoted the tumor-killing capacity of T cells *in vitro*. (A) Evaluation of the effects of IR-TAM@Alb on T cell killing to 5637 cells. 5637 cells were treated with TAM@Alb, IR-68@Alb, or IR-TAM@Alb for 24 h (calculated by TAM, IR-68, or IR-TAM concentration: 6 μM), followed by co-culture with activated T cells. (B) Living 5637 cells were quantified. Data were demonstrated as mean ± SD. *** *p* < 0.001.


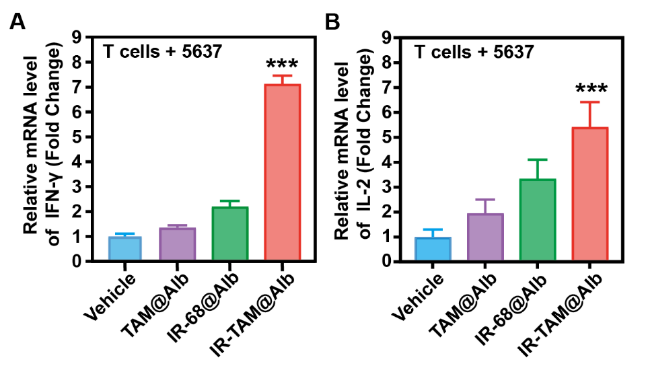


**Figure S14.** RT-q-PCR assay of IFN-γ and IL-2 in the supernatant of co-culture with T cells from different treatments. 5637 cells were treated with TAM@Alb, IR-68@Alb, or IR-TAM@Alb for 24 h (calculated by TAM, IR-68, or IR-TAM concentration: 6 μM), followed by co-culture with activated T cells. Data were demonstrated as mean ± SD. *** *p* < 0.001.


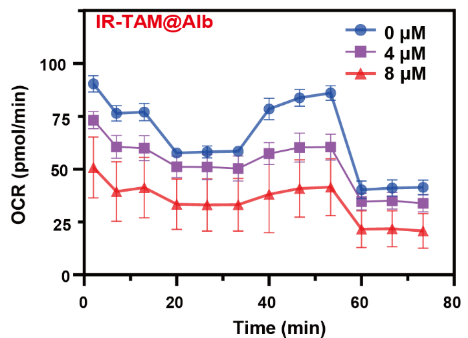


**Figure S15.** OCR (oxygen consumption rate) was used to evaluate the effect of different concentrations of IR-TAM@Alb (calaulated by IR-TAM concentration) on cellular oxygen consumption in 4T1 cells, n = 3. Data were demonstrated as mean ± SD.


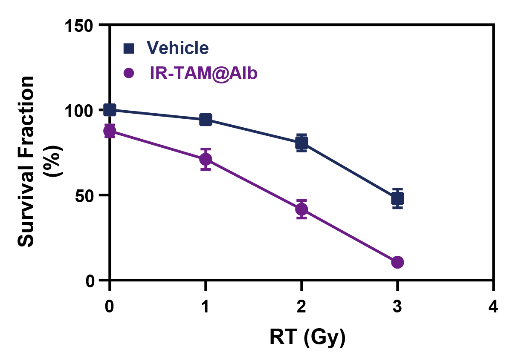


**Figure S16.** Evaluation of the sensitizting enhancement ratios (SER50) by colony formation assay treated by IR-TAM@Alb (calaulated by IR-TAM concentration: 6 μM) , n = 3. Data were demonstrated as mean ± SD.


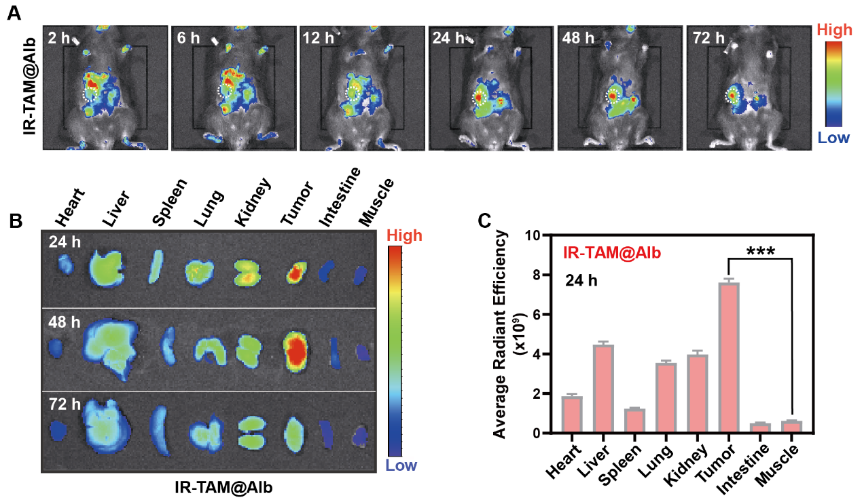


**Figure S17.** Evaluation of the tumor-targeting capacity of IR-TAM@Alb nanoparticles *in vivo*. (A) Near-infrared Ray (NIR) fluorescence images of MB49 tumor-bearing mice after the injection of IR-TAM@Alb (calculated by IR-TAM concentration: 2 mg/kg) intravenously at 2 h, 6 h, 12 h, 24 h, 48 h, and 72 h. (B) NIR fluorescence signals of major organs and tumors were collected from MB49 tumor-bearing mice, which received injections of IR-TAM@Alb (calculated by IR-TAM concentration: 2 mg/kg) at different time points. (C) Corresponding quantification of fluorescence signal from organs and tumors. Data were demonstrated as mean ± SD. *** *p* < 0.001.

**
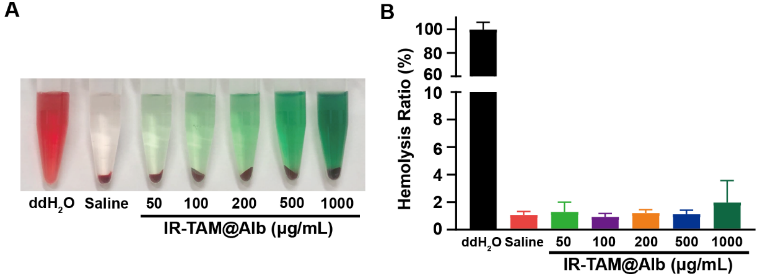
**

**Figure S18.** Hemolysis analysis of IR-TAM@Alb at various concentrations (calculated by IR-TAM concentration: 50, 100, 200, 500, 1000 μg/mL). Data were demonstrated as mean ± SD.

**
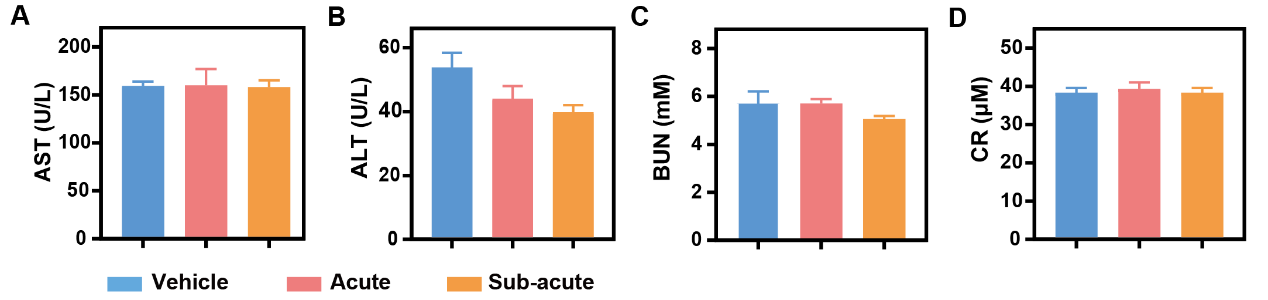
**

**Figure S19.** Evaluation of the acute toxicity and subacute toxicity of IR-TAM@Alb (calculated by IR-TAM concentration, 5 mg/kg) on BALB/C female mice by the liver and kidney function detection after 24 h or 14 days treatments (n = 3). Data were demonstrated as mean ± SD.


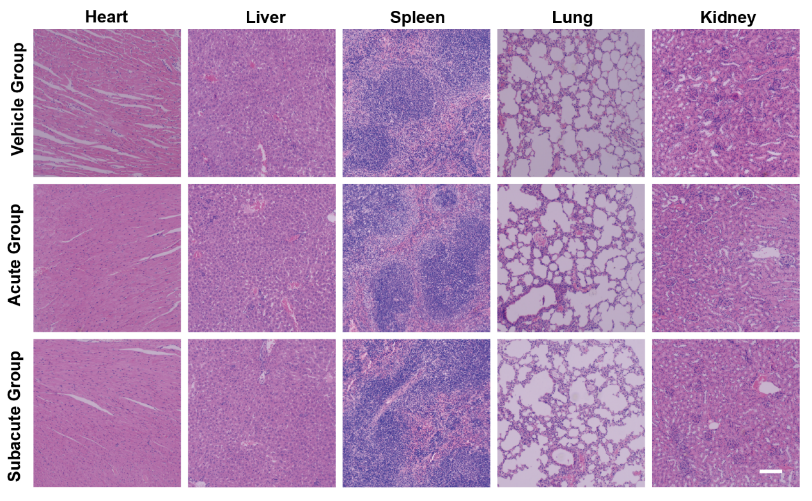


**Figure S20.** Representative H&E staining images of the major organs collected from healthy mice treated with either Vehicle or IR-TAM@Alb (calculated by IR-TAM concentration, 5 mg/kg) were obtained at acute (24 hours post-treatment) and sub-acute periods (14 days post-treatment), scale bar = 50 μm.


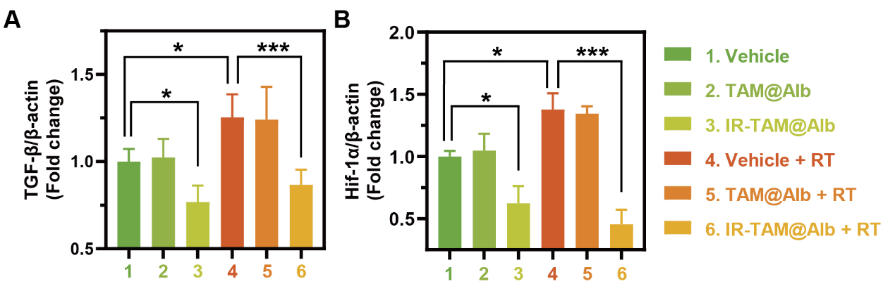


**Figure S21.** Detection of the expression levels of TGF-β and HIF-1α protein in tumors by western blotting assay in mice bearing 4T1 tumors that received various treatments and quantified using ImageJ software. Data were demonstrated as mean ± SD. * *p* < 0.05 and *** *p* < 0.001.

**
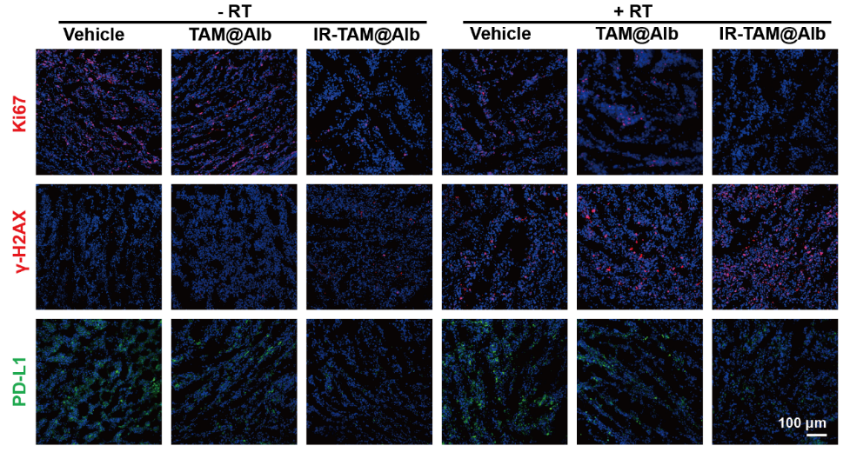
**

**Figure S22.** The immunofluorescence images of Ki67, γ-H_2_AX, and PD-L1 in 4T1 tumors after different treatments.


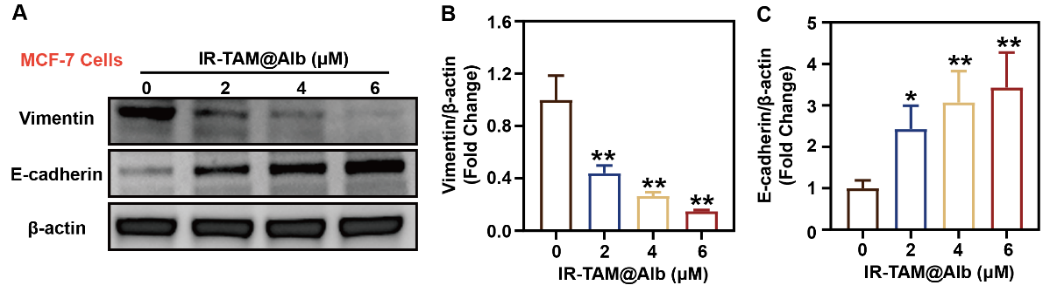


**Figure S23.** Detection of expression of Vimentin and E-cadherin protein in MCF-7 cells by western blotting assay that received various treatments and quantified using ImageJ software. Data were demonstrated as mean ± SD. * *p* < 0.05 and ** *p* < 0.01.


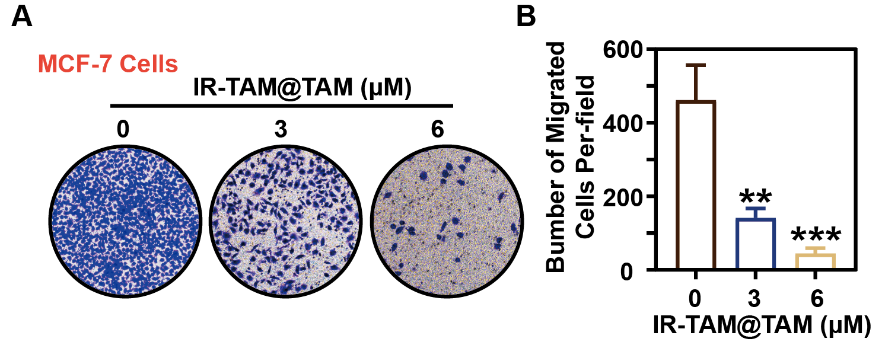


**Figure S24.** Evaluation of the migration of MCF-7 cells by the *in vitro* trans-well migration assay after IR-TAM@Alb treatment (calculated by IR-TAM concentration) (n = 3). Data were demonstrated as mean ± SD. ** *p* < 0.05 and *** *p* < 0.01.


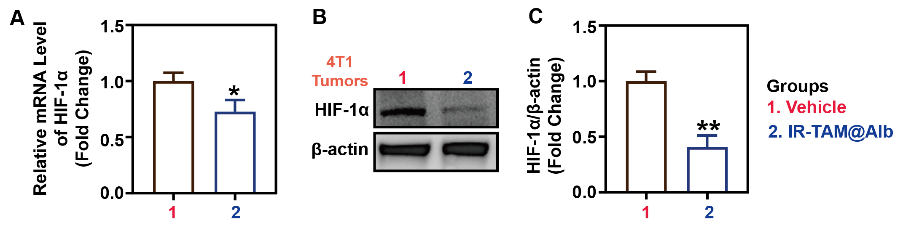


**Figure S25.** (A) RT-q-PCR assay of HIF-1α in 4T1 tumors after IR-TAM@Alb treatments. (B-C) Detection of expression of HIF-1α protein in 4T1 tumors by western blotting assay that received IR-TAM@Alb treatments and quantified using ImageJ software. Data were demonstrated as mean ± SD. * *p* < 0.05 and ** *p* < 0.01.


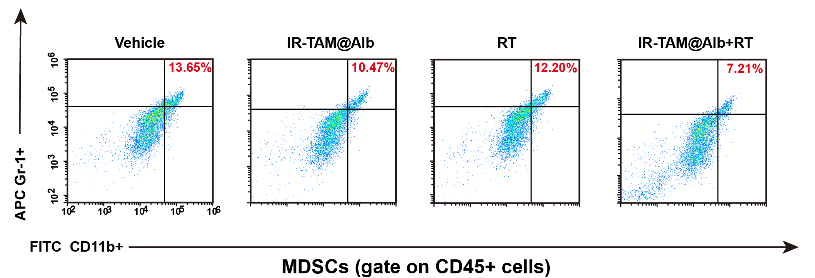


**Figure S26.** Analysis of CD11b^+^Gr-1^+^CD45^+^ MDSC cell populations in the mammary fat pad-implanted 4T1 tumors using flow cytometry (n = 5).


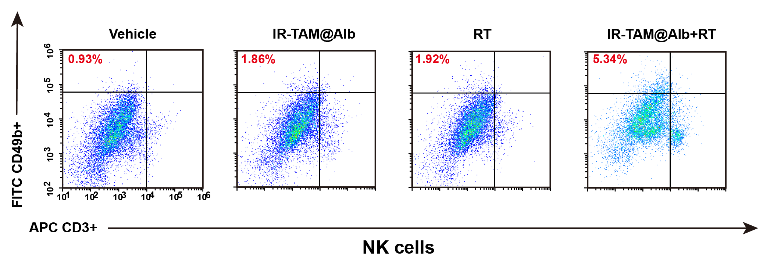


**Figure S27.** Analysis of CD3^-^CD49^+^ NK cell populations in the mammary fat pad-implanted 4T1 tumors using flow cytometry (n = 5).


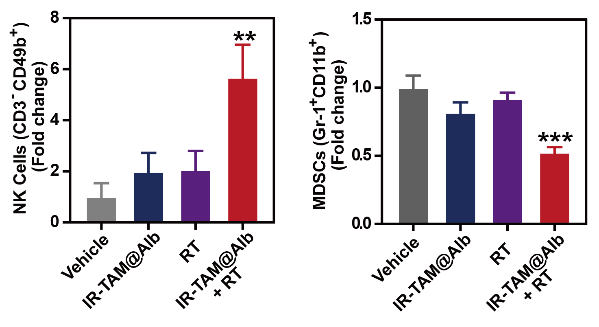


**Figure S28.** Quantification of the CD3^-^CD49^+^ NK cell and CD11b^+^Gr-1^+^CD45^+^ MDSC cell in the mammary fat pad implanted 4T1 tumors using flow cytometry (n = 5). Data were demonstrated as mean ± SD. ** *p* < 0.01 and *** *p* < 0.001.


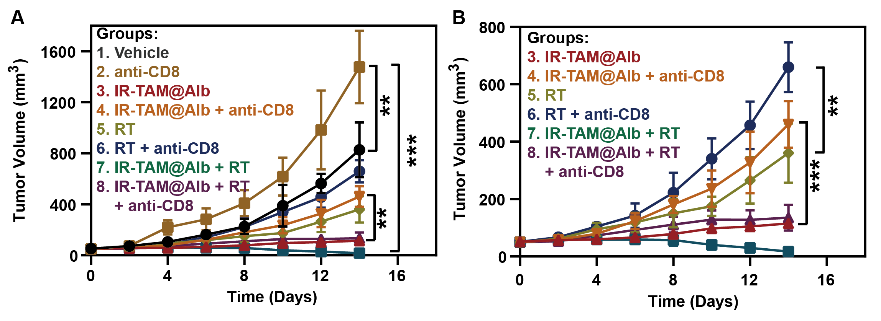


**Figure S29.** Growth curves of the mammary fat pad implanted 4T1 tumors (n = 5). Data were demonstrated as mean ± SD. ** *p* < 0.01 and *** *p* < 0.001.


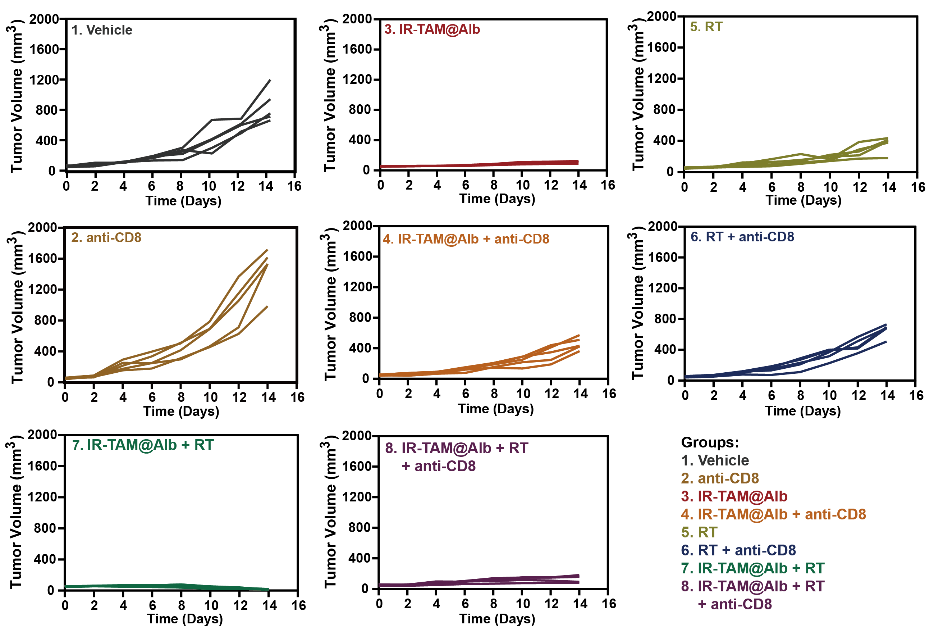


**Figure S30.** Growth curves of each Balb/C mice bearing the mammary fat pad implanted 4T1 tumors (n = 5).


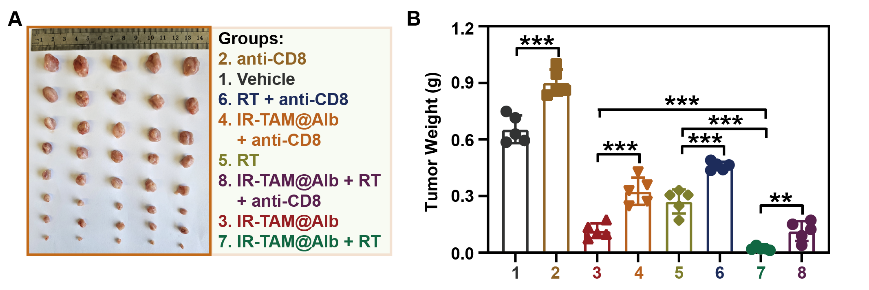


**Figure S31.** (A) The photograph depicting the mammary fat pad implanted 4T1 tumors collected on Day 14. (B) Tumor weight of the mammary fat pad implanted 4T1 tumors collected on Day 14. Data were demonstrated as mean ± SD. ** *p* < 0.01 and *** *p* < 0.001.

*
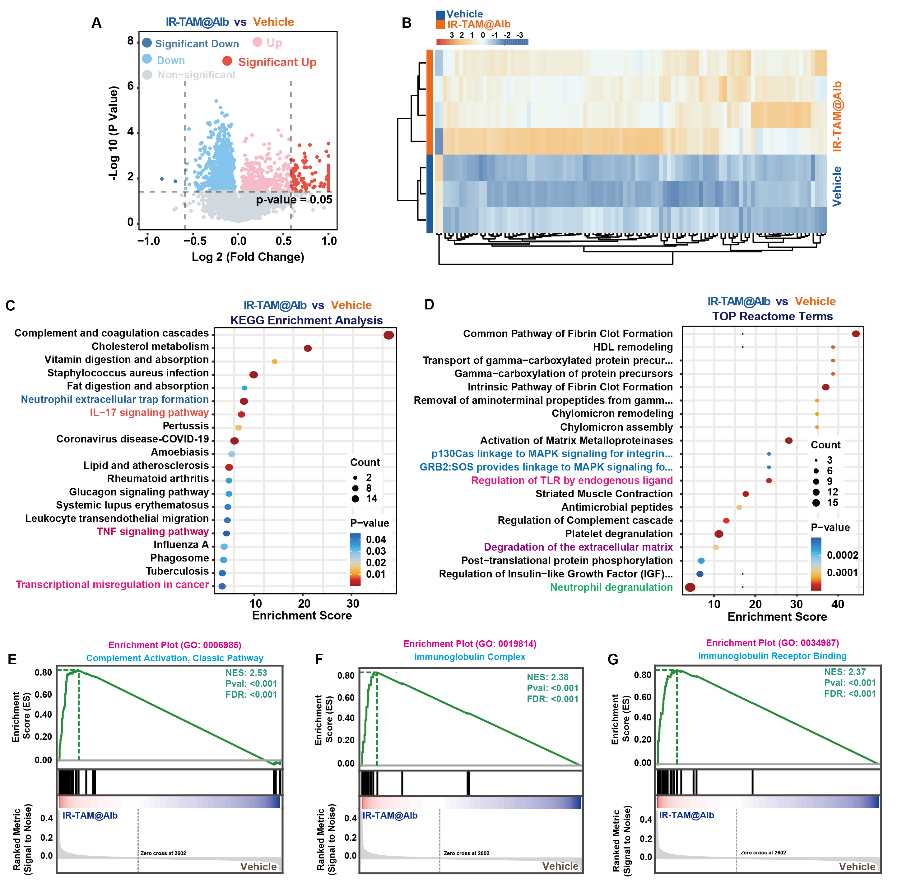
*

**Figure S32.** Proteomic analysis of IR-TAM@Alb-treated mammary fat pad implanted 4T1 tumors collected on Day 14. (A) Volcano plot based on fold change and significance of all proteins identified in the IR-TAM@Alb and Vehicle groups. (B) Heat map illustrating the differential expression of proteins in the mammary fat pad implanted 4T1 tumors collected on Day 14 following IR-TAM@Alb treatment or not. (C-D) KEGG enrichment analysis and TOP Reactome term enrichment analysis of differential pathways after IR-TAM@Alb treatment. (E-G) GO enrichment plots of differentially expressed proteins centralized in (E) complement activation, (F) immunoglobulin complex, and (G) immunoglobulin receptor binding.

**
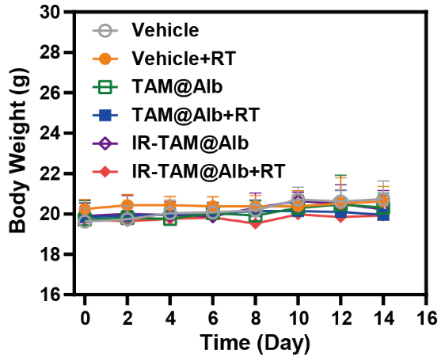
**

**Figure S33.** The body weight changes of MB49 tumor-bearing mice in the period from the start of treatment to surgery (n = 5). Data were demonstrated as mean ± SD.


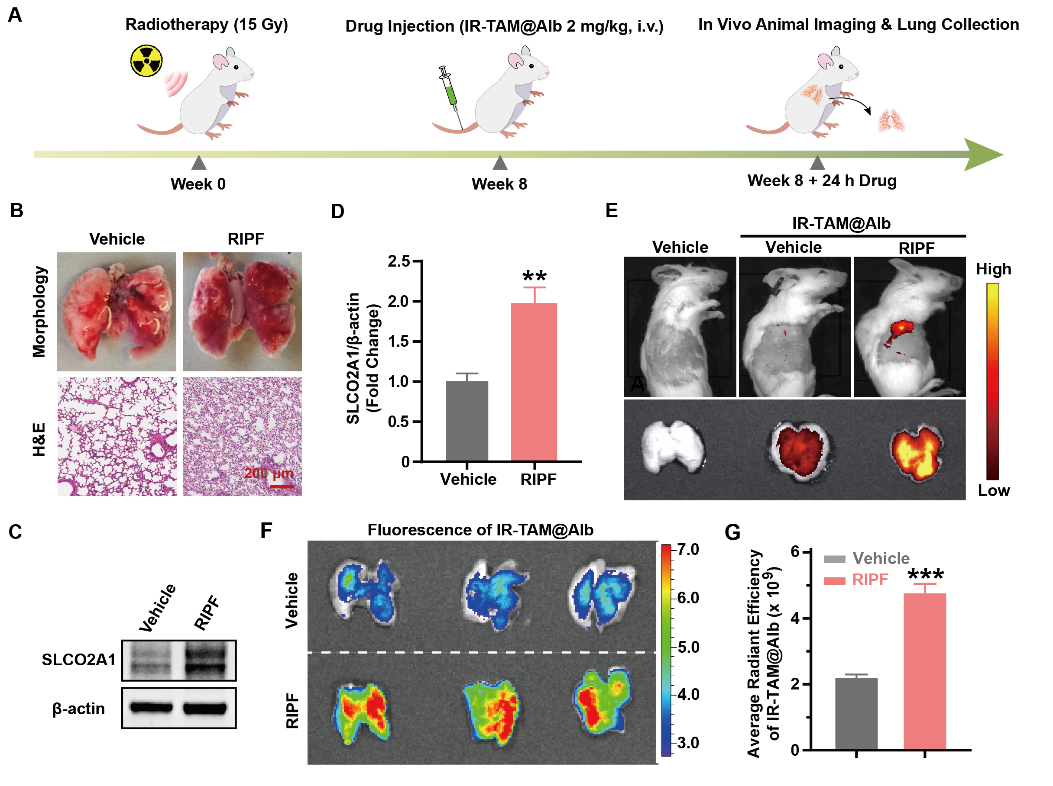


**Figure S34.** IR-TAM@Alb nanoparticles preferentially accumulated in the radiation-exposed lung tissue and alleviated RIPF. (A) Schematic diagram of the experimental scheme to evaluate the RIPF-targeting capacity of IR-TAM@Alb. (B) The representative images of morphology and H&E staining of lungs collected from mice received RT or not. (C-D) Detection of the expression of SLCO2A1 proteins in lungs received RT or not (n = 3). (E-F) NIR images of the whole bodies and the isolated lungs of mice with different treatments. (G) Quantification of the corresponding average radiant efficiency of IR-TAM@Alb in the lungs treated with RT or not (n = 3). Data were demonstrated as mean ± SD. ** *p* < 0.05 and *** *p* < 0.001.


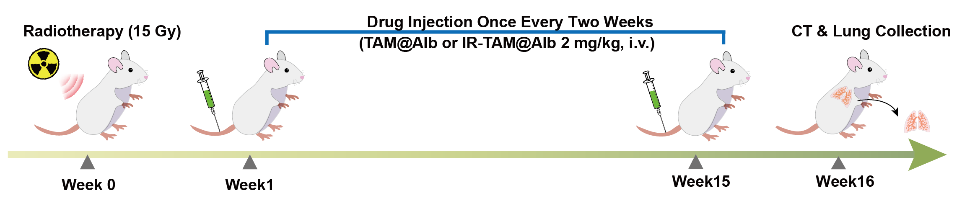


**Figure S35.** [Schematic diagram](javascript:;) of treatment and dosage regimen to evaluate the efficacy of IR-TAM@Alb in remising RIPF.


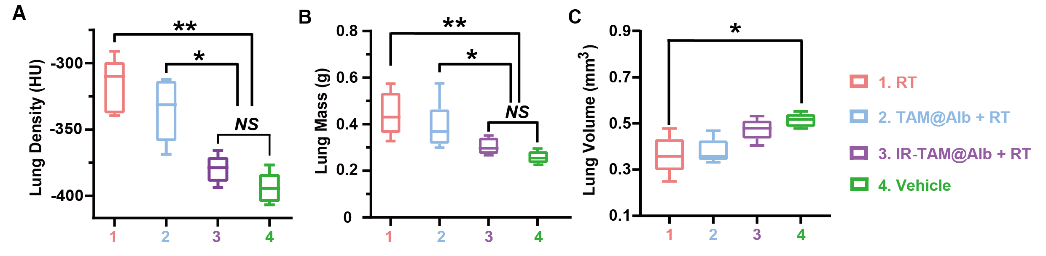


**Figure S36.** (A) Changes of the parenchymal lung density (Hounsfield unit, HU) after various treatments (n = 6). (B-C) Detection of the lung mass and lung volume after various treatments (n = 6). Data were demonstrated as mean ± SD. * *p* < 0.05 and ** *p* < 0.001. *NS* means no significant difference, compared with the vehicle group.

**
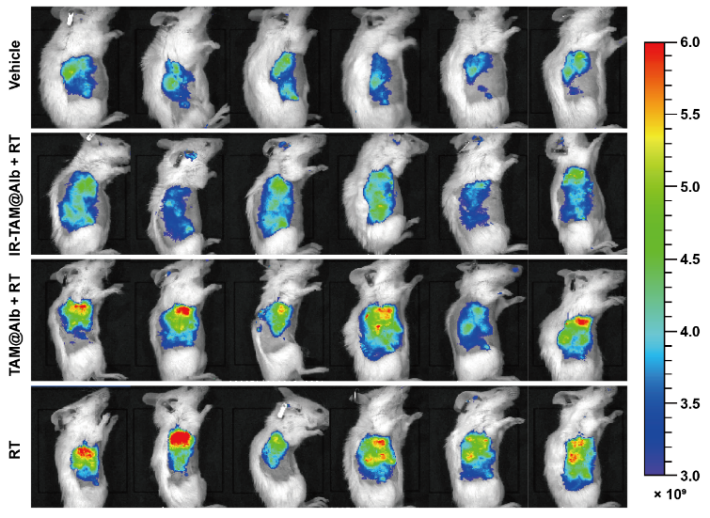
**

**Figure S37.** NIR imaging (detected by IR-TAM@Alb nanoparticles) of mice undergoing different treatments (n = 5). The samples were collected from mice that received whole-chest radiation and underwent the indicated treatment 16 weeks later.


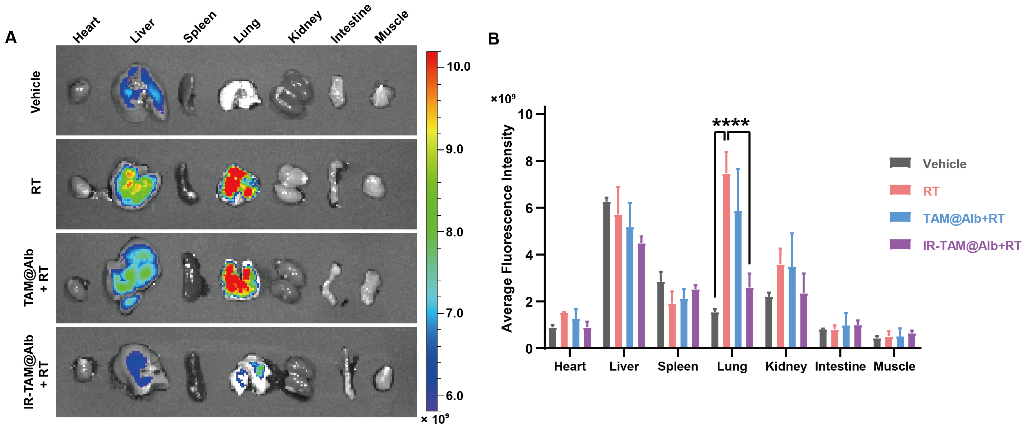
**Figure S38.** (A) NIR representative images of major organs were obtained 24 hours after intravenous injection of IR-TAM@Alb (calculated by IR-TAM concentration, 2 mg/kg) in mice following 16 weeks of various treatments. (B) Average fluorescence intensity was quantified for each group with the standardized method. Data were demonstrated as mean ± SD. ** *p* < 0.01.

**References:**

[1] Y. Liu, Z. Zhou, J. Hou, W. Xiong, H. Kim, J. Chen, C. Zheng, X. Jiang, J. Yoon, J. Shen, *Adv Mater* **2022**, e2206121.

[2] a) M. Li, Y. Shao, J. H. Kim, Z. Pu, X. Zhao, H. Huang, T. Xiong, Y. Kang, G. Li, K. Shao, J. Fan, J. W. Foley, J. S. Kim, X. Peng, *J Am Chem Soc* **2020**, *142* (11), 5380; b) C. M. Day, M. J. Sweetman, S. M. Hickey, Y. Song, Y. Liu, N. Zhang, S. E. Plush, S. Garg, *Molecules* **2021**, *26* (1); c) V. Sreekanth, S. Bansal, R. K. Motiani, S. Kundu, S. K. Muppu, T. D. Majumdar, K. Panjamurthy, S. Sengupta, A. Bajaj, *Bioconjug Chem* **2013**, *24* (9), 1468.

[3] a) X. Tan, S. Luo, L. Long, Y. Wang, D. Wang, S. Fang, Q. Ouyang, Y. Su, T. Cheng, C. Shi, *Adv Mater* **2017**, *29* (43); b) Y. Huang, J. Zhou, S. Luo, Y. Wang, J. He, P. Luo, Z. Chen, T. Liu, X. Tan, J. Ou, H. Miao, H. Liang, C. Shi, *Gut* **2018**, *67* (2), 307; c) Z. Zhou, J. Chen, Y. Liu, C. Zheng, W. Luo, L. Chen, S. Zhou, Z. Li, J. Shen, *Acta Pharmaceutica Sinica B* **2022**, *12* (11), 4204.

[4] X. Jiang, L. Yi, C. Li, H. Wang, W. Xiong, Y. Li, Z. Zhou, J. Shen, *ACS Nano* **2024**, *18* (4), 3331.

[5] L. Yi, X. Jiang, Z. Zhou, W. Xiong, F. Xue, Y. Liu, H. Xu, B. Fan, Y. Li, J. Shen, *Adv Mater* **2024**, e2304328.

[6] a) H. J. Lee, J. H. Ko, H. J. Kim, H. J. Jeong, J. Y. Oh, *JCI Insight* **2020**, *5* (12); b) A. Ajith, K. Mamouni, D. D. Horuzsko, A. Musa, A. K. Dzutsev, J. R. Fang, A. Chadli, X. Zhu, I. Lebedyeva, G. Trinchieri, A. Horuzsko, *J Clin Invest* **2023**, *133* (21); c) I. Dean, C. Y. C. Lee, Z. K. Tuong, Z. Li, C. A. Tibbitt, C. Willis, F. Gaspal, B. C. Kennedy, V. Matei-Rascu, R. Fiancette, C. Nordenvall, U. Lindforss, S. M. Baker, C. Stockmann, V. Sexl, S. A. Hammond, S. J. Dovedi, J. Mjosberg, M. R. Hepworth, G. Carlesso, M. R. Clatworthy, D. R. Withers, *Nat Commun* **2024**, *15* (1), 683; d) X. Qi, E. Cai, Y. Xiang, C. Zhang, X. Ge, J. Wang, Y. Lan, H. Xu, R. Hu, J. Shen, *Adv Mater* **2023**, *35* (48), e2306632.
